# Riboflavin-sensitized photoinduced degradation of donepezil hydrochloride: kinetic and computational insights for pharmaceutical formulation

**DOI:** 10.1039/d5ra07553j

**Published:** 2025-11-25

**Authors:** Tooba Khan, Zubair Anwar, Reem Altaf, Ayesha Awan, Aisha Noreen, Muneeba Usmani, Muhammad Ahsan Ejaz, Anam Khan, Sofia Ahmed, Muhammad Ali Sheraz, Toufeeque Ali

**Affiliations:** a Department of Pharmaceutical Chemistry, Baqai Institute of Pharmaceutical Sciences, Baqai Medical University Gadap Road, Superhighway Karachi–75340 Pakistan zubair_ana@hotmail.com zubair_ana@baqai.edu.pk; b Department of Pharmaceutical Chemistry, Faculty of Pharmacy, Capital University of Science and Technology Islamabad Expressway, Kahuta Road, Zone-V Islamabad Pakistan reem@cust.edu.pk; c Department of Pharmacognosy, Faculty of Pharmaceutical Sciences, Riphah International University G-7/4, 7th Avenue Islamabad Pakistan; d Department of Pharmaceutics, Baqai Institute of Pharmaceutical Sciences, Baqai Medical University Gadap Road, Superhighway Karachi–75340 Pakistan; e Indus Pharma Pvt. Ltd Plot 26,27 & 65 Sector 27 Korangi Industrial Area Karachi Pakistan; f Liaquat University Hospital Jamshoro Pakistan

## Abstract

Donepezil (DPZ) photosensitized degradation by riboflavin (RF) at pH 2.0–12.0 has been carried out in aerobic and anaerobic conditions under UV and visible irradiation to give an idea to pharmaceutical formulators to develop a stable co-formulation to administer to elderly patients with neurodegenerative diseases (Alzheimer's disease, Parkinson's disease). The photolysis rate constants (*k*_obs_) in aerobic and anaerobic conditions in the presence of RF (0.1–0.5 × 10^−4^ M) range from 0.25–11.5 and 0.025–1.120 × 10^−2^ min^−1^, respectively. RF catalyzes the photodegradation of DPZ, and the second-order (*k*_2_) constants in aerobic and anaerobic conditions range from 0.32 to 8.22 and 0.032 to 0.805 × 10^−2^ M^−1^ min^−1^, respectively, indicating that with an increase in the concentrations of RF, the rate of photolysis of DPZ also increases. The *k*-pH profile shows a bell-shaped curve from pH 2.0 to 4.0 and from pH 5.0 to 12.0, forming a sigmoid curve. The two-component spectrometric and HPLC green methods have been developed and validated for estimating DPZ and RF in pure and degraded solutions. Additionally, computational studies have been conducted to assess the formation of the ground-state complex, binding affinity, and molecular interactions between DPZ and RF.

## Abstract 2

Schematic representation of riboflavin-induced photodegradation of donepezil under aerobic and anaerobic conditions, illustrating the photosensitized pathways leading to the formation of photodegradation products.

## Graphical abstract

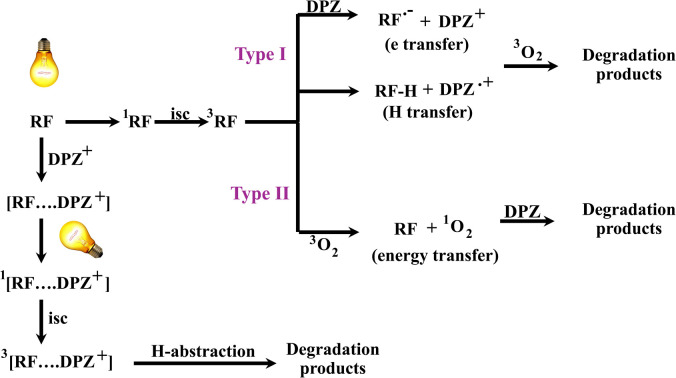

## 1 Introduction

**Fig. 1:**
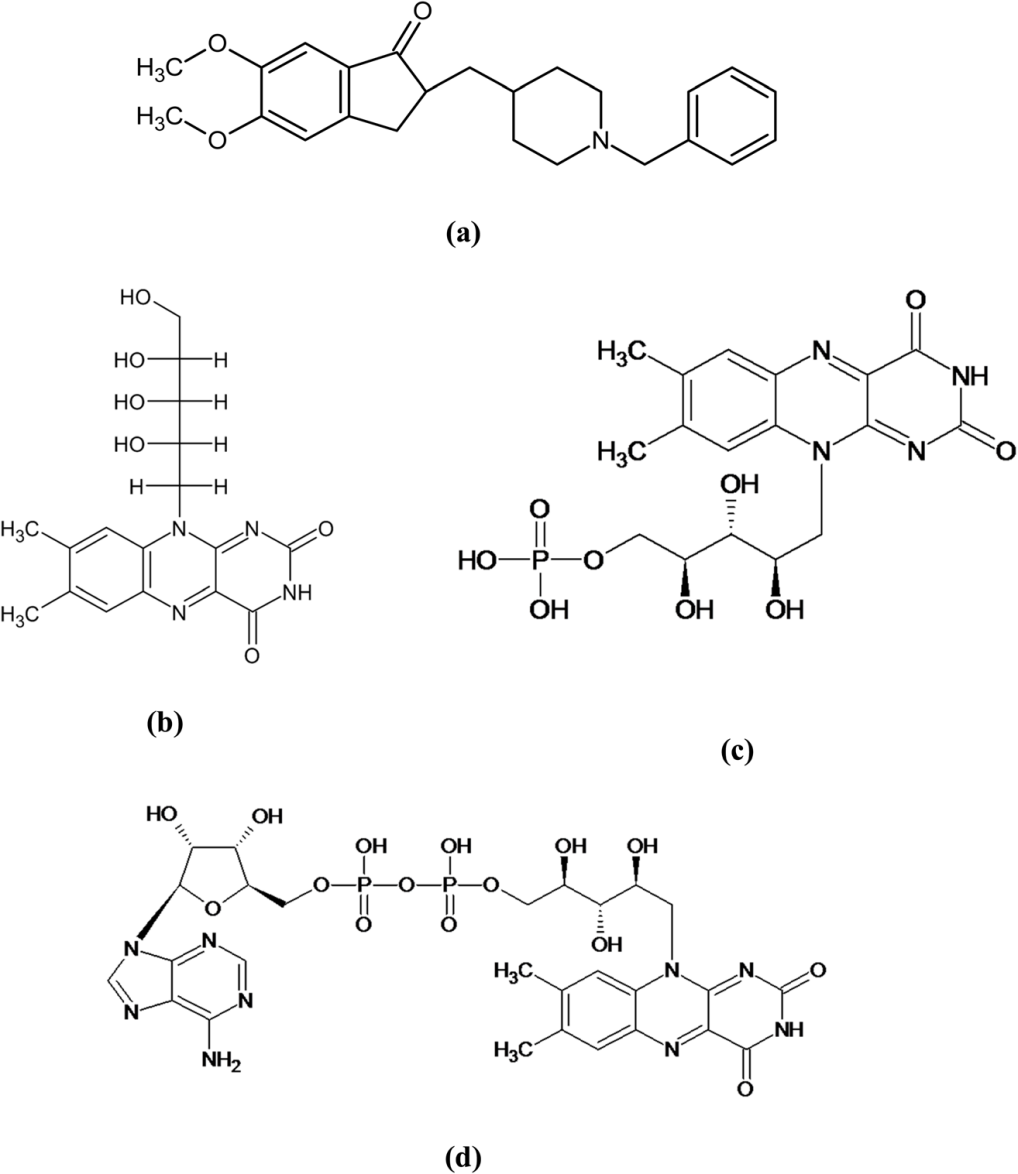
Chemical structures of donepezil (DPZ, (a)), riboflavin (RF, (b)), and its coenzymes (FMN (c), FAD (d)).

The stability (chemical, physical, and photo) of drugs and drug products is an important attribute that needs to be evaluated to predict their potency, efficacy, safety, expiry, and shelf life. Whenever the drugs and drug substances are irradiated with artificial UV or visible light, it leads to the photodegradation of these compounds, resulting in the loss of therapeutic activity and the formation of degradation products.^1–6^ Donepezil (2,3-dihydro-5,6-dimethoxy-2-[[1-(phenylmethyl),-4-piperidinyl] methyl]-1*H*-inden-1-one) (DPZ) (Fig. 1a) is a piperidine derivative that is an acetylcholine esterase inhibitor^7–10^ that is used for the treatment of dementia, Alzheimer's disease, and Parkinson's disease.^7–19^

**Fig. 2:**
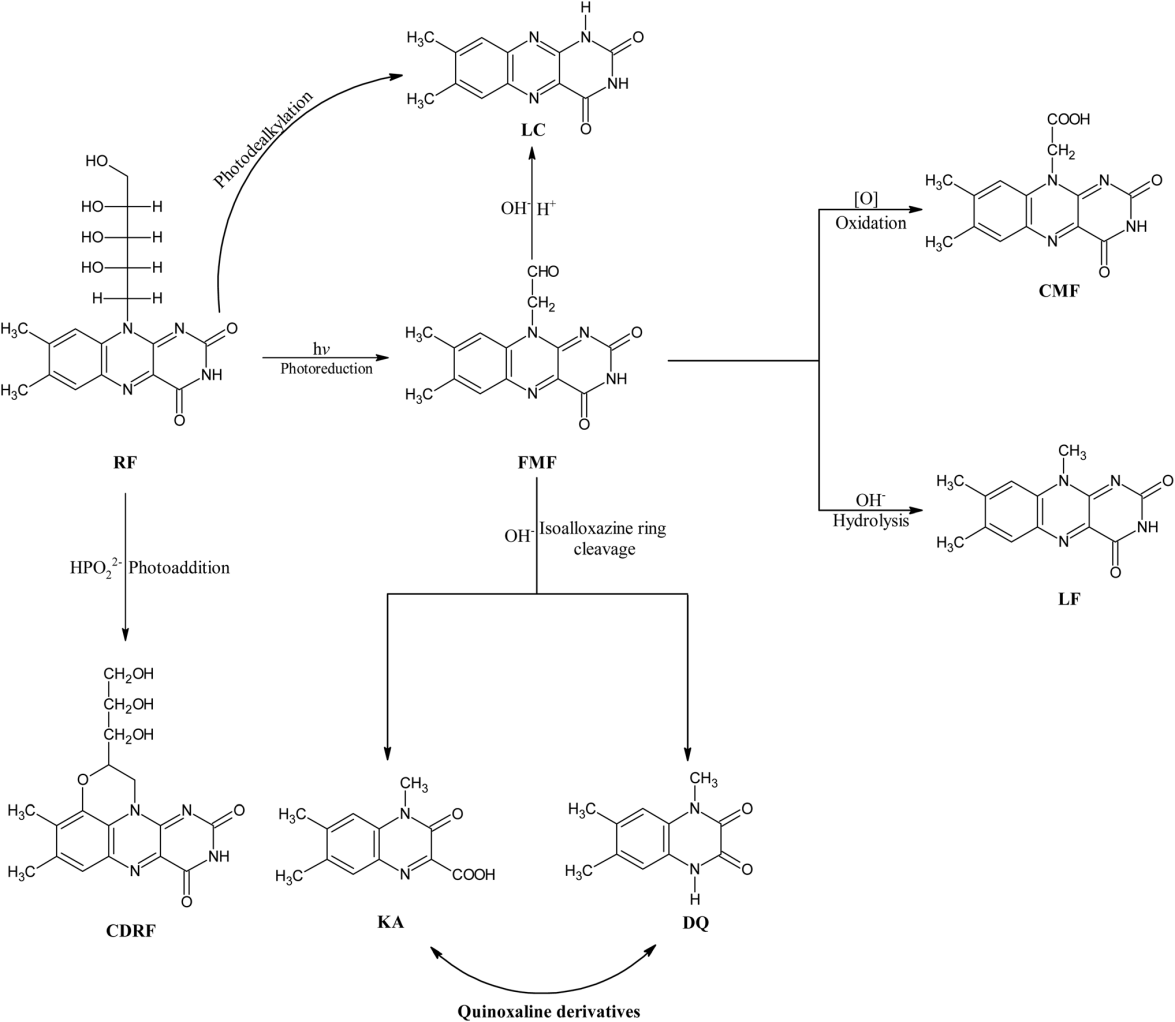
Photodegradation pathways (photodealkylation, photoreduction, photooxidation, photoaddition) of RF photodegradation to form its photoproducts (FMF, CMF, LF, CDRF, KA, DQ).

Riboflavin (vitamin B_2_, RF) (7,8-dimethyl-10-[(2S,3S,4R)-2,3,4,5-tetrahydroxypentyl] benzo[g]pteridine-2,4-dione) (Fig. 1b) is a precursor of flavin coenzymes (flavin mononucleotide (FMN) (Fig. 1c), flavin dinucleotide (FAD) (Fig. 1d))^20,21^ in a biological system acting as electron carriers in oxidation–reduction reactions. RF is a photosensitive compound, and its photochemistry is of great interest to scientists.^22–35^ It degrades into several photoproducts, which include formylmethylflavin (FMF), lumichrome (LC), lumiflavin (LF), cyclodehydroflavin (CDRF), carboxymethylflavin (CMF), 1,2-dihydro-1-methyl-2-keto-3-quinoxaline carboxylic acid (KA), and 1,2,3,4-tetrahydro-1-methyl-2,3-dioxo-quinoxaline (DQ) (Fig. 2) *via* photoreduction, photodealkylation, photooxidation, and photoaddition pathways.^36–49^ RF, when irradiated with light (visible, UV), converts into excited singlet and triplet states and is involved in the photodegradation pathways to form photodegraded products ((LC, CDRF *via* excited singlet state) and (FMF, LC, LF, CMF, KA, DQ *via* excited triplet state).^37,40–42,45–47,50–56^ It is a well-known photosensitizer, and the excited triplet state of RF interacts with molecular oxygen (^3^O_2_) and substrate (*i.e.*, 2,4-dinitrophenol, aflatoxin B_1_, bilirubin, ascorbic acid, indole, unsaturated fatty acids, amino acids, pyridoxine, *etc.*)^48,49,57–94^ to carry forward photosensitization characteristics. This photosensitization catalysis property of RF has been widely used for photodynamic inactivation (PDI) of microbes^95–97^ and parasites,^98–102^ and photodynamic therapy.^103–108^

Vitamins (*e.g.*, RF, thiamine, cyanocobalamin, niacinamide, pyridoxine) are often used by elderly patients, and DPZ is used in these patients for the treatment of Alzheimer's disease, dementia, and Parkinson's disease.^109–111^ These two drugs (RF and DPZ) are often co-administered in patients, which may lead to a photochemical interaction or RF-sensitized photodegradation of DPZ, resulting in loss of potency and the formation of degradation products that may be toxic. Therefore, there is a dearth of literature, as no such study has been carried out to date. Therefore, the objective of the present investigation is to evaluate the effect of RF and its concentrations on the photodegradation of DPZ in both aerobic and anaerobic conditions, within the pH range of 2.0–12.0, under UV and visible irradiation. The two-component spectrometric and HPLC methods have been developed and validated for the simultaneous quantification of DPZ and RF in pure and degraded solutions.

Additionally, the greenness, blueness, and redness of these methods have been evaluated using the national environmental method index (NEMI), analytical eco-scale, green analytical procedure index (GAPI), blue applicability grade index (BAGI), red analytical performance index (RAPI), and the AGREE calculator. These two developed analytical methods are also statistically compared for the evaluation of accuracy and precision in estimating RF and DPZ when co-formulated for administration to elderly patients to treat vitamin deficiency and neurodegenerative diseases. Also, computational studies have been carried out to evaluate the complex formation between RF and DPZ and their binding affinity. The proposed study also provides insight into maintaining the stability of DPZ using a rate-pH profile, which helps select the optimum pH for co-formulating both drugs. Mechanistic photodegradation or photochemical interaction pathways are also proposed in both aerobic and anaerobic conditions. The proposed study would be beneficial for pharmaceutical scientists in preparing a stable co-formulation of both drugs. If they are co-administered together, it is expected that this approach will not cause any toxic or adverse effects on the biological system.

## 2 Materials and methods

### 2.1 Materials

DPZ HCl (≥98.0%) and RF (99.0%) were procured from Sigma-Aldrich (St. Louis, USA). Solvents used in this present study have been purchased from Merck & Co. (White House Station, NJ, USA) and were of HPLC grade. Freshly prepared double reverse osmosis deionized water with zero total dissolved solids (TDS) was used throughout the study (FineTech Water Treatment, Pakistan). The filtration of solutions and solvents was carried out using a Millipore filtration assembly. The buffer solutions used in this study are KCl–HCl (pH 1.0–2.0), citric acid-Na_2_HPO_4_ (pH 2.5–8.0), Na_2_B_4_O_7_–HCl (pH 8.5–9.0), Na_2_B_4_O_7_–NaOH (pH 9.5–10.5), and Na_2_HPO_4_–NaOH (pH 11.0–12.0). The ionic strength in each case was 0.005 M.

### 2.2 Precautions

The freshly prepared solutions of DPZ, alone and in the presence of RF, were prepared in the dark to prevent any chemical or photochemical changes. All the photolysis reactions of DPZ and RF have been carried out under subdued light.

### 2.3 Methods

#### 2.3.1 Purity confirmation

The purity confirmation of analytes (*i.e.*, DPZ, RF) is a crucial consideration before developing and validating analytical methods and conducting photodegradation studies, as it ensures that no impurities or degradation products are present in the crude sample, which could lead to erroneous results. Therefore, FTIR spectroscopy and differential scanning calorimetry (DSC) were used to confirm the purity of the samples, and details are given in the supplementary data section.

#### 2.3.2 pH measurements

The pH of DPZ alone and DPZ and RF solutions was determined using an LCD-display Elmetron pH meter (Model CP-501, with a sensitivity of ±0.01 units). Commercially available buffer tablets with pH levels of 4.0, 7.0, and 9.0 were used for a calibration of the pH meter.

#### 2.3.3 Thin-layer chromatography (TLC)

DPZ and RF, TLC of pure and photodegraded solutions were performed using pre-coated 250 µm silica gel (GF254, Merck). The solvent systems used are water: acetic acid: 1-butanol: 1-propanol (18 : 2:50 : 30, v/v),^112^ and 1-butanol: 1-propanol: acetone: water: glacial acetic acid (28.5 : 28.5 : 14.3 : 14.3 : 14.3, v/v).^113^

#### 2.3.4 UV-visible spectroscopy

The spectral and absorbance measurements of the pure and degraded solutions of DPZ, as well as the DPZ and RF solutions, were determined using a UV-visible Spectrometer (Thermo Scientific, Evolution 201, USA) in quartz cells with a 1 cm path length.

#### 2.3.5 Measurements of light intensities

The light intensity measurements of Philips 30W TUV and HPLN 125W lamps were carried out using potassium ferrioxalate actinometry, as proposed by Hatchard and Parker.^114^ The values obtained were 2.31 ± 0.10 × 10^17^ and 5.51 ± 0.22 × 10^18^ q s^−1^ for Philips 30 W TUV and HPLN 125 W lamps, respectively.

#### 2.3.6 Estimation of quantum yields of photolysis

The photolysis quantum yields (*Φ*) of DPZ in the presence of RF (0.50 × 10^−4^ M) were calculated using the values of light intensity (*Q*) from Philips 30W TUV and HPLN 125 W lamps. The values of *Φ* have been obtained by calculating the area under the emission bands of Philips 30 W TUV and HPLN 125 W lamps absorbed by DPZ, divided by the total area of the emission bands of these two lamps.1
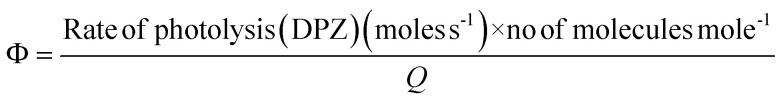


#### 2.3.7 Two-component spectrometric assay method

The two-component spectrometric assay method for the simultaneous determination of DPZ and RF has been carried out at 325 and 445 nm, respectively, at pH 7.0 (phosphate buffer, 0.005 M). A 1 mL sample from the pure and photodegraded solutions was pipetted out into a volumetric flask (Pyrex), to which phosphate buffer (pH 7.0, 0.005 M) was then added to make up the volume. The proposed two-component spectrometric method for the simultaneous estimation of DPZ and RF has been validated according to the guidelines of the International Council on Harmonization (ICH).^115^

#### 2.3.8 HPLC assay method

The HPLC assay method for the simultaneous determination of DPZ and RF was developed using an HPLC System (Model LC10–ADV, Shimadzu, Japan) equipped with a PDA detector (Model SPD-10A, VP) and a controller (Model SCL–10AVP), which is connected to a microcomputer. The assay procedure was carried out using a C18 column (5 µm thickness, 4.6 × 250 mm diameter, Welch, Zhejiang, China) with a solvent system of acetonitrile: KH_2_PO_4_ (0.005 M) (38 : 62, v/v) at a flow rate of 1.2 mL min^−1^. The pH of the solvent system was maintained at 2.5, and the detection was carried out at a wavelength of 264 nm. A 20 µl glass syringe was used to inject the sample taken from the pure and degraded solutions. The proposed HPLC method for the simultaneous quantification of DPZ and RF has been developed and validated following the ICH guidelines.^115^

#### 2.3.9 Photolysis

DPZ (1.00 × 10^−4^ M) alone and in the presence of RF (0.10–0.50 × 10^−4^ M) solutions were prepared at pH 2.0–12.0. These prepared solutions were continuously bubbled with air and nitrogen to maintain the aerobic and anaerobic conditions, respectively. These solutions were then irradiated with UV and visible irradiation in a specific chamber (Fig. S1a and b). The temperature of these solutions was maintained at 25 ± 1 °C by placing them in a water bath. The UV and visible spectral emissions are shown in Fig. S2a and b, which display emission at 270 nm (maximum emission) and 319, 375, 405, 494, 555, and 578 nm (minimum emission) for the UV irradiation lamp. However, the visible irradiation lamp shows emissions at 340, 360, 395, 490, 555, 590, 620, and 700 nm (maximum emission) and 580 and 700 nm (minimum emission). The air-prone oxidation of DPZ was evaluated by placing the control solutions in the dark. At appropriate time intervals, the samples were withdrawn and subjected to TLC, UV-visible spectrometric, and HPLC analysis.

#### 2.3.10 Method validation

The two-component spectrometric and HPLC assay methods for the simultaneous determination of DPZ and RF were validated by the ICH Guidelines.^115^ The validation parameters include linearity, limit of detection (LOD), limit of quantification (LOQ), accuracy, precision, and robustness. The details of the studied parameters are given in the SI Section.

#### 2.3.11 Statistical evaluation

A comparison of the results obtained from the two-component spectrometric and HPLC assay methods for the simultaneous quantification of DPZ and RF was carried out using the Statistical Package for the Social Sciences (SPSS, version 25.0).

#### 2.3.12 Computational studies

##### 2.3.12.1 Quantum chemical calculations

Quantum chemical calculations, including molecular orbital analysis, electrostatic potential (ESP) mapping, Mulliken atomic charge calculations, and HOMO–LUMO gap analysis, were conducted to understand the polarizability and electron distribution characteristics of the complex in both neutral and deprotonated states. All quantum chemical calculations were performed using Gaussian09 software.^116^ The DPZ in complex with RF was first drawn using Avogadro software. Geometry optimizations were later performed using HF/6-31G(d) level of theory. Electronic properties, including Mulliken charges, frontier molecular orbitals (HOMO–LUMO), energies of HOMO–LUMO, and molecular electrostatic potential (MEP) surfaces, were calculated. GaussView was used to visualize the results of quantum mechanical calculations. The frontier molecular orbitals (FMO) were examined in order to examine the electron density distribution in the protonated and deprotonated forms of the DPZ–RF complex. The effect of pH was simulated by deprotonation of RF and subsequent optimization of the complex.

##### 2.3.12.2 Molecular docking analysis

The molecular interaction and formation of the ground-state complex between DPZ and RF were determined using *in silico* studies. The formation of bonds and binding affinity (kcal mol^−1^) between DPZ and RF was also evaluated to determine how strongly they are bonded to each other. The structures of DPZ and RF were taken from PubChem and Biovia Discovery Studio to determine the binding affinity, molecular interaction, and the formation of the ground-state complex. The Pyrex virtual screening software was used to dock DPZ with RF.

#### 2.3.13 Total organic carbon (TOC) analysis

The Sievers 500 RL online TOC analyzer was used to evaluate the mineralization of DPZ, RF, and DPZ-RF for their photodegradation. The solutions of DPZ (1.00 × 10^−4^ M), RF (0.50 × 10^−4^ M), and their mixture (DPZ–RF) at pH 7.0 were analyzed before and after irradiation to estimate the TOC content in the prepared solutions.

## 3 Results and discussion

### 3.1 Confirmation of purity

Prior to the method development, validation, and degradation studies, the purity confirmation is an important consideration. Therefore, we conducted FTIR and DSC to evaluate the purity of DPZ and RF in the pure samples used in this study.

DPZ (2,3-dihydro-5,6-dimethoxy-2-[[1-(phenylmethyl)-4-piperidinyl]methyl]-1*H*-inden-1-one hydrochloride (Fig. S3a) shows peaks for –CH aromatic bending, C–C stretching, C–N stretching, –CH aliphatic bending, –CH_2_ bending, C=C (aromatic), C=O, C–H aliphatic stretching, –CH_2_ aliphatic stretching, and C–H aromatic stretching at 702, 1071, 1081, 1262, 1376, 1453, 1589, 1697, 2849, 2923, and 3008 cm^−1^, respectively (Fig. S3a).^117^ Chemically, RF is 7,8-dimethyl-10-[(2S,3S,4R) −2,3,4,5-tetrahydroxypentyl]benzo[g]pteridine-2,4-dione, and its FTIR spectrum is given in Fig. S3b. It shows peaks for OH/NH, C=O, C=C, C=N, C–OH, and ribose moiety at 3370, 1650, 1580, 1550, and 1850 cm^−1^, respectively.^48,49,118^ These spectra of DPZ and RF, which were obtained, are compared with the reported ones. It has been found that the powder samples of DPZ and RF used in this study do not contain any impurities or degraded products, making them suitable for method development, validation, and photodegradation studies.

The purity of DPZ and RF was further confirmed using DSC. DPZ shows an endothermic peak at 234 °C (Fig. S4).^16,119,120^ In contrast, RF decomposed at 280 °C and does not show any endothermic peak.^48,49,118^ Therefore, the results obtained indicated that the powder samples of DPZ and RF are pure and free from degradation products and can be used for the method development, validation, and degradation studies.

### 3.2 DPZ and RF spectral characteristic

The absorption spectra of DPZ and RF were measured at pH 2.0 and 7.0. DPZ exhibits absorption maxima at 209, 225, 269, and 325 nm at both pH 2.0 and 7.0, as previously reported.^16,121^ In comparison, RF exhibits absorption maxima at 223, 267, 385, and 445 nm at pH 2.0 and 7.0.^48,49,122^ The absorption spectra showing absorption maxima for DPZ (1.00 × 10^−4^ M) and RF (0.50 × 10^−4^ M) at pH 2.0 and 7.0 are given in Fig. S5a and b. There is a considerable difference between the absorption maxima of DPZ (325 nm) and RF (445 nm) at pH 2.0 and 7.0. Therefore, pH 7.0 has been used for the two-component spectrometric method for the simultaneous quantification of DPZ and RF. At pH 7.0, wavelengths of 325 and 445 nm were used to develop and validate the two-component spectrometric methods for DPZ and RF, respectively, following the ICH guidelines.^115^

### 3.3 Assay methods

#### 3.3.1 Two-component spectrometric method

**Fig. 3:**
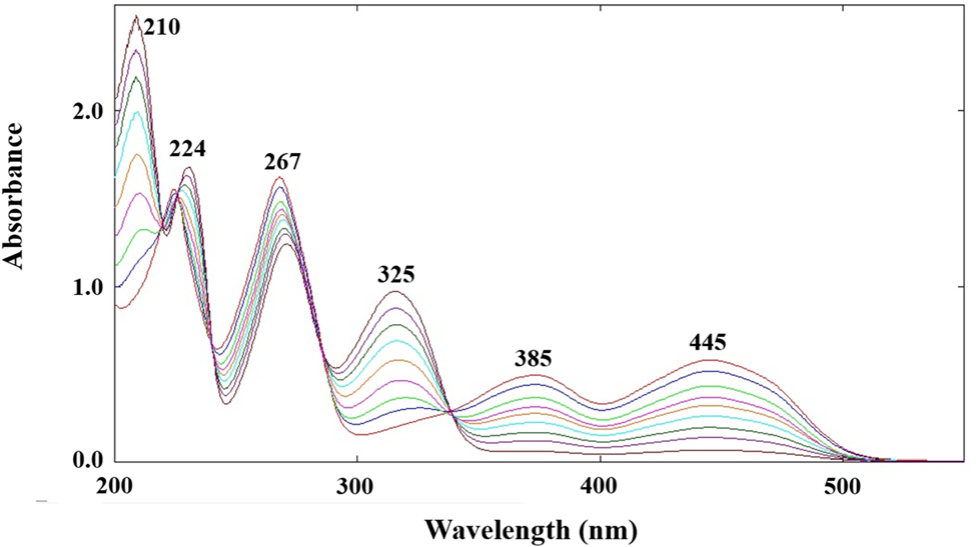
DPZ (0.10–0.90 × 10^−4^ M) and RF (0.05–0.45 × 10^−4^ M) overlay absorption spectra of synthetic mixtures at pH 7.0.

DPZ and RF show absorption maxima at 209, 225, 269, and 325 and 223, 267, 385, and 445 nm, respectively (Fig. S5a and b). At pH 7.0, there is a considerable difference between the absorption maxima of DPZ (325 nm) and RF (385 and 445 nm). Therefore, this pH has been selected for the development and validation of a two-component spectrometric method for determining DPZ and RF in pure and degraded solutions, as per the ICH guidelines.^115^ The proposed two-component spectrometric method is found to be linear in the concentration ranges of 0.10–1.0 and 0.05–0.50 × 10^−4^ M for DPZ and RF, respectively (Fig. S6a). The validation statistical data, calculated from the linearity, are given in Table 1. The accuracy and precision of the two-component spectrometric method for the simultaneous quantification of DPZ and RF have been determined and are given in Tables S1 and S2, respectively. The accuracy and precision (%RSD) for the estimation of DPZ and RF are in the ranges of 100.0–100.1 and 99.9–100.3%, respectively, and 0.05–1.00% and 0.10–0.21%, respectively. Additionally, the robustness of the proposed method has been evaluated, and it was found that the process remains robust for determining DPZ and RF even after applying slight variations to the procedure. The results of the robustness are found to be in the recovery (%) range of 99.9–100.2% and 100.0–101.2% for DPZ and RF, respectively. The accuracy, precision, and reproducibility of the proposed method have been evaluated by applying it to a synthetic mixture of DPZ and RF (Fig. 3). The results obtained for the synthetic mixture, used for the simultaneous determination of DPZ and RF, are presented in Table 2. It has been found that the proposed method is accurate, precise, and reproducible for the simultaneous quantification of DPZ and RF.

**Table 1:** Two-component spectrometric method validation data for the simultaneous determination of donepezil (DPZ) and riboflavin (RF) at pH 7.0

	PD	RF
*λ*_max_, nm	325	445
Linearity		
Range (M ×10^4^)	0.10–1.00	0.05–0.50
Correlation coefficient	0.9998	0.9999
Slope (M × 10^4^)	1.14	1.15
SE of slope (M × 10^2^)^a^	0.59	0.27
Intercept	0.00007	−0.00411
SE of intercept (×10^2^)^b^	0.41	0.19
SD of intercept (×10^2^)^c^	1.28	0.60
Mean accuracy (%) ± SD	100.0 ± 0.93	100.1 ± 0.73
Precision (%RSD)	0.93	0.72
LOD (M ×10^5^)^d^	0.37	0.17
LOQ (M ×10^5^)^e^	1.12	0.52

a Standard error of slope.

b Standard error of intercept.

c Standard deviation of intercept.

d Limit of detection.

e Limit of quantification.

**Table 2:** Analysis of DPZ and RF in synthetic mixtures using the developed simultaneous two-component spectrometric method

DPZ				RF			
Added (M × 10^4^)	Found (M × 10^4^)^a^	Recovery (%)	RSD (%)	Added (M × 10^4^)	Found (M × 10^4^)^a^	Recovery (%)	RSD (%)
0.100	0.099	99.0	0.22	0.450	0.448	99.6	0.10
0.200	0.199	99.5	0.41	0.400	0.399	99.8	0.19
0.300	0.300	100.0	0.36	0.350	0.349	99.7	0.26
0.400	0.401	100.3	0.15	0.300	0.299	99.7	0.41
0.500	0.502	100.4	0.72	0.250	0.251	100.4	0.62
0.600	0.599	99.8	0.29	0.200	0.201	100.5	0.74
0.700	0.703	100.4	0.11	0.150	0.152	101.3	0.63
0.800	0.802	100.3	0.21	0.100	0.100	100.0	0.82
0.900	0.900	100.0	0.58	0.050	0.051	102.0	0.14

a *n* = 5.

#### 3.3.2 HPLC assay method

**Fig. 4:**
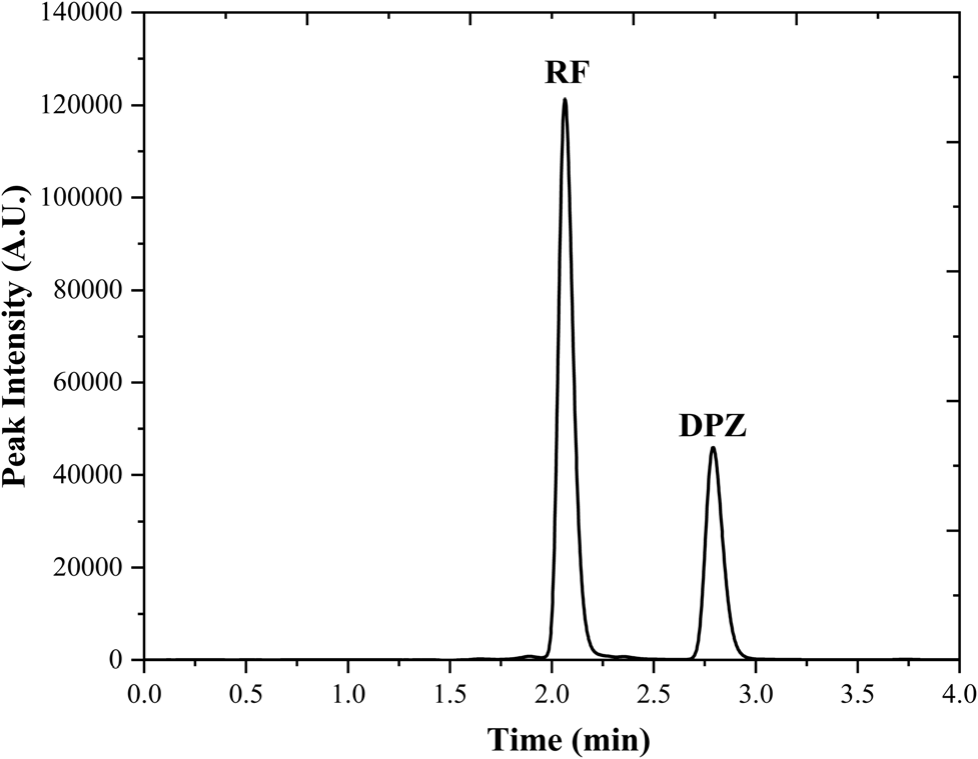
RF (0.05 × 10^−4^ M) and DPZ (1.00 × 10^−4^ M) chromatograms at pH 2.5, where RF and DPZ peaks are at 2.06 and 2.78 min, respectively.

An HPLC assay method for the simultaneous determination of DPZ and RF has been developed and validated according to the ICH guidelines.^115^ The chromatograms for the simultaneous estimation of DPZ (1.00 × 10^−4^ M) and RF (0.50 × 10^−4^ M) are given in Fig. 4. This developed HPLC method is linear in the concentration of 0.10–1.0 and 0.05–0.50 × 10^−4^ M for DPZ and RF, respectively (Fig. S6b). The linearity data have been used for the statistical evaluation, and the results are given in Table 3. Also, the accuracy and precision of the proposed HPLC method have been determined, and it was found that the accuracy and precision are in the range of 99.7–100.0%, 99.7–99.9% and 0.07–0.08%, and 0.20–0.29% for DPZ and RF, respectively (Tables S3 and S4). The robustness of the proposed HPLC method for the simultaneous estimation of DPZ and RF was evaluated by deliberately varying the method parameters, and the results obtained are presented in Table S5. The % recovery for the DPZ and RF is in the range of 100.2–101.4% and 99.8–101.4%, respectively. Additionally, the accuracy (%recovery) and precision (%RSD) of the HPLC method were evaluated by preparing synthetic mixtures of DPZ and RF (Table S6). The % recovery and % RSD were found to be in the ranges of 99.7–100.5% and 98.0–100.3%, and 0.17–0.87% and 0.18–0.99%, respectively, for DPZ and RF.

**Table 3:** An HPLC method validation data for the simultaneous determination of donepezil (DPZ) and riboflavin (RF) at pH 2.5

	DPZ	RF
*λ*_max_, nm	264	264
Retention time (*t*_R_), min	2.78	2.06
RSD (%)	0.45	0.33
N^a^	8425	9852
T^b^	0.45	0.65
Linearity		
Range (M ×10^4^)	0.10–1.00	0.05–0.50
Correlation coefficient	0.9998	0.9999
Slope (M × 10^10^)	0.85	2.02
SE of slope (M × 10^4^)^c^	0.49	0.29
Intercept (×10^3^)	−2.72	4.94
SE of intercept (×10^4^)^d^	0.34	0.20
SD of intercept (×10^4^)^e^	1.10	0.63
Mean accuracy (%) ± SD	100.2 ± 0.85	100.2 ± 0.65
Precision (%RSD)	0.84	0.65
LOD (M ×10^5^)^f^	0.43	0.10
LOQ (M ×10^5^)^g^	1.29	0.31

a Theoretical plates.

b Tailing factor.

c Standard error of slope.

d Standard error of intercept.

e Standard deviation of the intercept.

f Limit of detection.

g Limit of quantification.

Also, these two developed two-component spectrometric and HPLC methods for the simultaneous quantification of DPZ (0.10–1.00 × 10^−4^ M) in aqueous solution (Table 4) have been statistically compared. The Student's *t*-test has been applied to the results obtained from UV spectrometric and HPLC methods for the determination of DPZ in the presence of RF, and the calculated *t* value is 0.062 with a *p* value of 0.952, which is greater than 0.05 (*p* value), indicating that there is no significant difference between these two developed methods. This shows that the results obtained from these two methods in pure and degraded solutions are reliable, accurate, precise, robust, and comparable.

**Table 4:** Comparative analysis for the determination of DPZ using two developed methods (UV and HPLC)

UV				HPLC		
Added (M × 10^4^)	Found^a^ (M × 10^4^)	Recovery (%)	RSD (%)	Found^a^ (M × 10^4^)	Recovery (%)	RSD (%)
0.100	0.100	100.0	0.22	0.099	99.0	0.52
0.200	0.199	99.5	0.32	0.201	100.5	0.11
0.300	0.301	100.3	0.56	0.300	100.0	0.63
0.400	0.400	100.0	0.48	0.402	100.5	0.42
0.500	0.502	100.4	0.15	0.499	99.8	0.39
0.600	0.599	99.8	0.69	0.602	100.3	0.85
0.700	0.701	100.1	0.72	0.700	100.0	0.69
0.800	0.798	99.8	0.22	0.801	100.1	0.14
0.900	0.903	100.3	0.19	0.899	99.9	0.36
1.000	0.998	99.8	0.95	1.000	100.0	0.47

a UV-visible spectrometric and high-performance liquid chromatographic method.

#### 3.3.3 Evaluation of greenness and blueness of the methods

The greenness, blueness, and redness of the developed analytical methods are crucial for ensuring environmental protection, analyst safety, and cost-effectiveness.^123–125^ Therefore, the greenness, blueness, and redness of the developed and validated analytical methods (two-component and HPLC) have been evaluated using NEMI, the analytical eco-scale, GAPI, the AGREE calculator, RAPI, and BAGI tools.

**Fig. 5:**
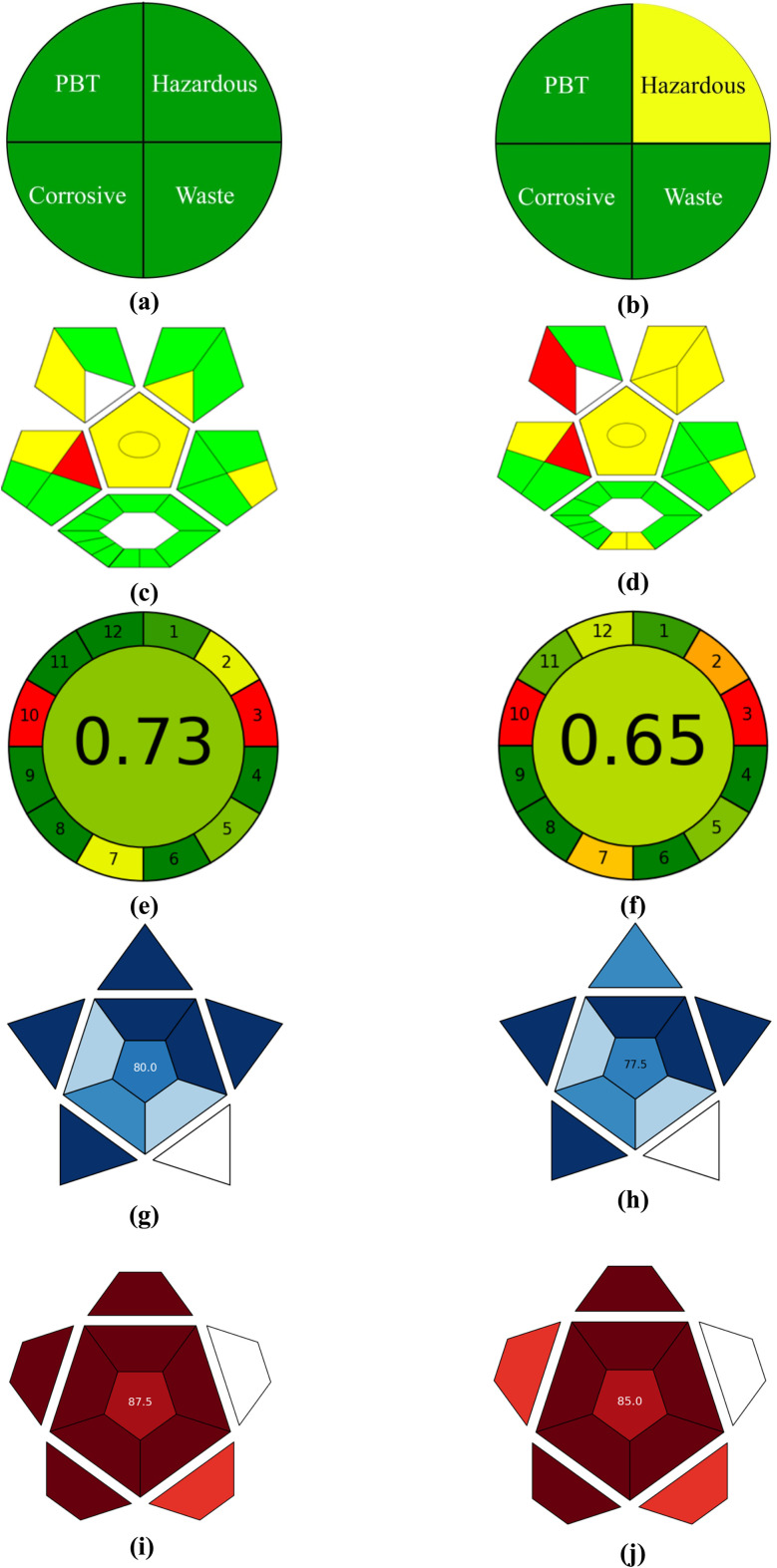
The pictograms for the analysis of greenness, blueness, and redness of the two-component spectrometric (a, c, e, g, i) and HPLC (b, d, f, h, j) methods for the simultaneous determination of DPZ and RF. Where a and b = NEMI; c and d = ComplexGAPI; e and f = AGREE; g and h = BAGI; and i and j = RAPI pictograms.

The NEMI illustrates the greenness determination of the developed methods and is presented in Fig. 5a and b. NEMI is a four-quadrant figure that depicts the use of persistent, bioaccumulative, and toxic (PBT) materials, hazardous chemicals, and the generation of waste materials (>50 g).^126,127^ The obtained pictogram for the two-component spectrometric method indicates that all four quadrants are green (Fig. 5a), suggesting that the process is environmentally friendly. However, in the HPLC pictogram, three quadrants are green, and one is yellow, which is due to the use of acetonitrile (38.0 mL/100 mL) in the mobile phase. At the same time, three quadrants are green because the materials used are not persistent, bioaccumulative, toxic, or corrosive. The pH of the mobile phase was 2.5, which is in the acceptable range (2.0–12.0), and the generated waste is less than 50 g.

The analytical eco-scale is another tool for calculating the environmental impact of analytical methods, which is dependent on the calculation of penalty points based on the use of hazardous chemicals, energy consumption, reagent amounts, and the production of waste materials.^127^ If the analytical eco-scale scores were greater than 75, it is considered excellent green. Whereas if scores are 20–75 or less than 50, the method is said to be considerably green or not green, respectively. The penalty points (PPs) for two-component spectrometric and HPLC methods for the quantification of DPZ and RF have been calculated and subtracted from the total value (100) to obtain the analytical eco-scale score. The PPs for the spectrometric and HPLC methods are 6.00 and 19.0, with a total analytical eco-scale score of 94.0 and 81.0, respectively (Table 5). This indicates that the developed methods are excellent in terms of greenness (analytical eco-scale score >75), as stated by the Globally Harmonized System of Classification and Labelling of Chemicals.^128^

**Table 5:** Analytical eco-scale determination of the two developed methods (UV and HPLC)

	Penalty points	
	UV-vis spectroscopy	HPLC
Citro-phosphate buffer (pH 7.0, 0.002 M)	0.00	0.00
Acetonitrile	—	6.00
Instrument energy consumption (kWh)	1.00	2.00
Sonicator	0.00	0.00
Occupational hazards	0.00	6.00
Waste (>10.0 mL)	5.00	5.00
Totally penalty points	6.00	19.0
**Analytical eco-scale score** a	94.0	81.0

a UV-visible spectrometric and high-performance liquid chromatographic method.

The GAPI (green analytical procedure index) is another tool used to assess the environmental sustainability of analytical methods. It is the combination of the analytical eco-scale score and NEMI to determine the greenness of the analytical methods. The GAPI pictogram consists of 15 subsections, each represented by green, yellow, and red colours, which correspond to excellent, considerably green, and non-green methods.^129^ Five pentangles consist of 1 to 15 subsections, each showing green, yellow, and red colours, which helps in predicting the greenness of the analytical methods. However, if any subsection is not applicable, that subsection remains white or uncoloured. The pre-analysis process is presented as an additional hexagonal section, which resembles analytical eco-scale parameters.^130^ The complex GAPI pictograms for two-component spectrometric methods and HPLC assay methods for the simultaneous determination of DPZ and RF are shown in Fig. 5c and d. The two-component GAPI pictogram (Fig. 5c) shows 8 greens, 4 yellows, 1 red, and 1 uncoloured, indicating that the developed method is green. However, in the case of the HPLC method, 6 greens, 5 yellows, 2 reds, and 1 uncoloured indicate that it is a considerably green method.^131,132^ Additionally, in the case of UV, all hexagonal subsections are green. In contrast, for the HPLC method, except for two that are yellow, all are green, indicating that the UV method is green and the HPLC method is considerably greener.

The AGREE calculator gives a cumulative score based on 12 parameters. If the cumulative score is 1.0, then the method is considered excellent green. However, a cumulative score below 0.60 indicates that the method is regarded as a non-green method. The AGREE calculator figure obtained for the proposed methods is presented in Fig. 5e and f, with a cumulative score of 0.73 (Fig. 5e) and 0.65 (Fig. 5f) for the two-component spectrometric and HPLC assay methods, respectively, for the simultaneous quantification of DPZ and RF. The AGREE calculator figure indicates that the two-component spectrometric method is excellent, whereas the HPLC method is considerably green.

Also, the practical applicability of the developed method has been evaluated by the blue applicability grade index (BAGI). There are 10 attributes (type of analysis, mixture analysis, sample preparation, analytical technique, number of samples that can be simultaneously treated, number of samples analyzed per hour, type of materials and reagents, requirement of preconcentration, degree of automation, and the amount of sample) based on which the practical applicability of the method is determined. The BAGI evaluation depends on four scores: 2.5, 5.0, 7.5, and 10.0, which correspond to white, light blue, blue, and dark blue, respectively.^133^ This overall assessment of the analytical method yields an asteroid pictogram featuring a number in the center, ranging from 25 to 100. If the value is 25, then the method's performance in terms of applicability is the worst. Whereas, if the value is 60, the method is practical in terms of applicability, and if the value is 100, the method exhibits excellent performance in terms of practical applicability.^133^ The asteroid pictogram for the two-component and HPLC methods is shown in Fig. 5g and h. The overall scores for the two-component and HPLC methods are 80.0 (Fig. 5g) and 77.5 (Fig. 5h), respectively, indicating that both methods possess good practical applicability and can be easily applied for the simultaneous quantification of DPZ and RF, as well as for real-time analysis.

Red analytical performance index (RAPI) is another tool like BAGI, which is based on the measurements of 10 attributes (linearity estimation, working range, limit of quantification (LOQ), accuracy, precision (repeatability, intermediate), recovery and matrix effect, robustness, selectivity).^134^ These parameters were selected from the ICH guidelines for the method validation.^115,133–139^ The assessment of these attributes is conducted on a five-level scale, ranging from 0 to 10, where 0 represents the worst result and 10 represents the best result for the developed and validated method.^134^ The sustainability of the developed methods (two-component spectrometric and HPLC) has been determined using the RAPI tool (Fig. 5i and j). The overall scores for the two-component spectrometric and HPLC methods for the simultaneous quantification of DPZ and RF are 87.5 (Fig. 5i) and 85.0 (Fig. 5j), respectively. These scores indicate that these methods possess excellent analytical performance, characterized by a good working range, accuracy, precision, robustness, and % recovery of the analyte, with a % RSD of less than 1.0%.

### 3.4 Spectral variations in photolyzed solutions

**Fig. 6:**
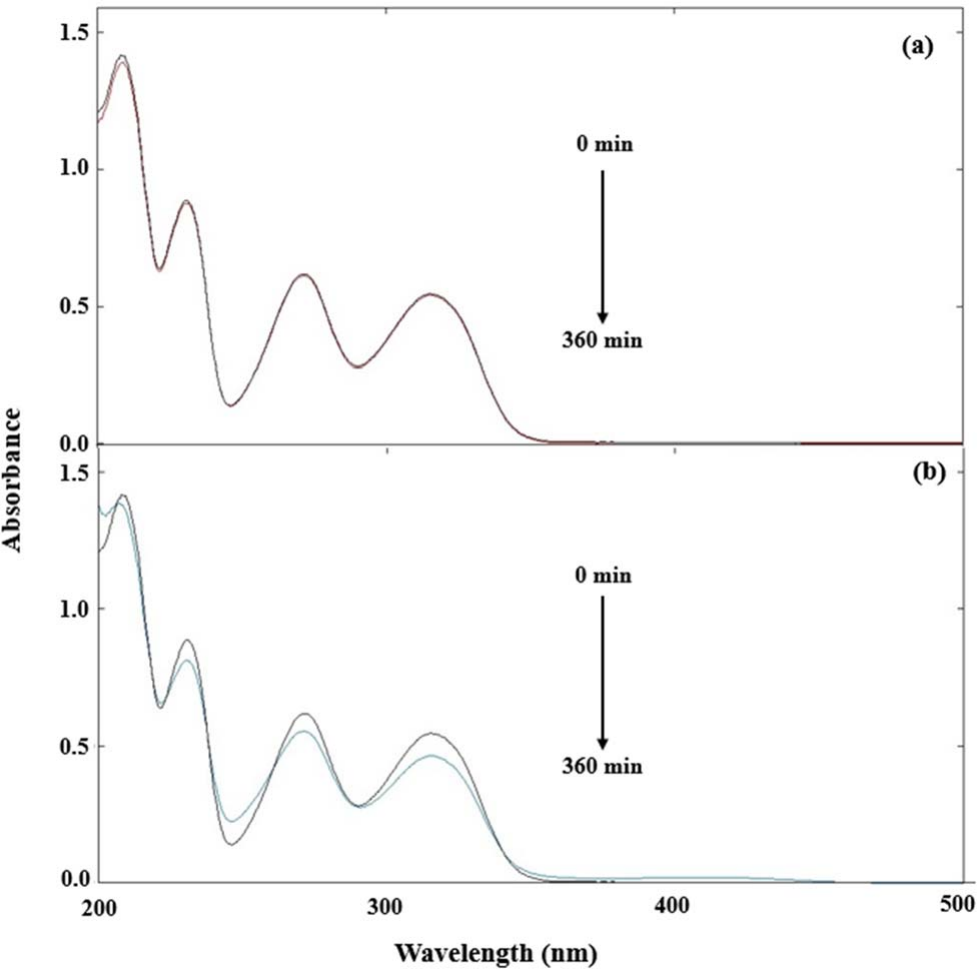
Absorption spectra of DPZ (1.00 × 10^−4^ M) before and after visible (a) and UV irradiation (b). Where the black and red lines represent before (0 min) and after (360 min) photolysis.

DPZ shows absorption maxima at 209, 225, 269, and 325 nm (Fig. S5). At pH 7.0, DPZ (1.00 × 10^−4^ M) solutions were exposed to UV and visible irradiation. Overlay spectra for the photolysis of DPZ are given in Fig. 6a and b. The extent of change in the spectra of DPZ is higher in the case of UV irradiation (Fig. 6b) as compared to that of visible irradiation (Fig. 6a). The UV and visible irradiation sources used in this study show emission at 270 nm (maximum emission), 319, 375, 405, 494, 555, and 578 nm (minimum emission), and 340, 360, 395, 490, 555, 590, 620, and 700 (maximum emission) and 580 and 700 (minimum emission). The change in absorbance at 360 min is higher in the case of UV irradiation (Fig. 6b) due to the higher emission at 253 and 325 nm, which corresponds to the absorption maximum of DPZ, resulting in higher light absorption and, consequently, higher photodegradation of DPZ. However, in the case of visible irradiation, the spectral change is low or negligible (Fig. 6a), which is due to its emission at wavelengths greater than 350 nm. Therefore, DPZ absorbs a minimum of visible light, resulting in low or minimal degradation in the case of visible irradiation (Fig. 6a).

**Fig. 7:**
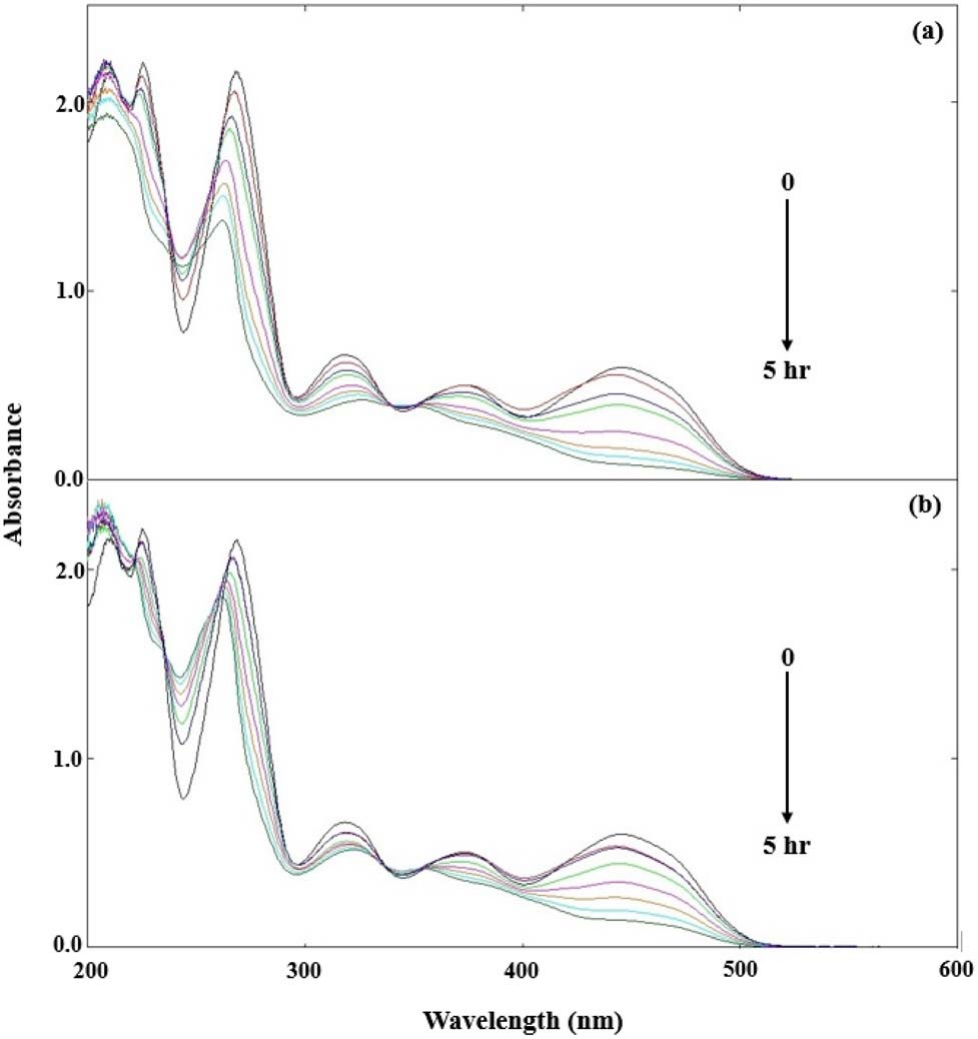
Absorption spectra of DPZ (1.00 × 10^−4^ M) in the presence of RF (0.50 × 10^−4^ M) under UV (a) and visible (b) irradiation at intervals of 0 to 5 h at pH 7.0.

The spectral/absorbance changes at pH 7.0 in a mixture of DPZ (1.00 × 10^−4^ M) and RF (0.50 × 10^−4^ M) have also been determined under UV and visible irradiation. RF is known to be a potent sensitizer, and this sensitization process is primarily mediated by the 385 and 445 nm bands of RF.^48,49,140^ The overlay spectra of DPZ (1.00 × 10^−4^ M) and RF (0.50 × 10^−4^ M) under UV and visible irradiation are given in Fig. 7a and b. When DPZ is in the presence of RF that is irradiated under UV and visible light, a loss of absorbance at 325 nm has been observed, which corresponds to the absorption maximum of DPZ. The loss of absorbance is found to be higher in the case of visible irradiation (Fig. 7b) than that of UV irradiation (Fig. 7a). The loss of absorbance at 325 nm is higher in visible irradiation due to higher absorption of visible light by RF (>350 nm), which corresponds to its absorption maxima of 385 and 445 nm that are involved in the photosensitization process, resulting in the higher degradation of DPZ. However, the loss of absorbance at 325 nm is less in UV irradiation due to its maximum emission at 254 nm, which does not correspond to the absorption maxima of RF (385 and 445 nm) responsible for photosensitized degradation. Therefore, the energy transfer in the case of UV irradiation from RF to DPZ is lower, resulting in a decrease in degradation. Whereas this energy transfer from RF to DPZ is higher in the case of visible irradiation, which results in higher degradation of DPZ. The rates of photodegradation of DPZ in the presence of RF under visible irradiation are higher than those of UV irradiation. RF shows strong absorption in the visible region (*λ*_max_ 385 and 445 nm). When RF is excited, it converts into the excited singlet state, which *via* isc converted into the excited triplet state. The triplet lifetime is 0.3–0.6 and 15–20 µs in aerobic and anaerobic conditions,^141,142^ respectively, with an energy of 42–46 kcal mol^−1^,^143–145^ making it an effective energy donor. However, DPZ does not significantly absorb visible irradiation but can accept energy from the triplet state of RF *via* triplet–triplet energy transfer (TTET) or electron transfer, which results in the increased degradation of DPZ in the presence of RF under visible irradiation, as reported earlier in the case of ascorbic acid^47^ and pyridoxine.^48^

### 3.5 DPZ and RF stoichiometry

**Fig. 8:**
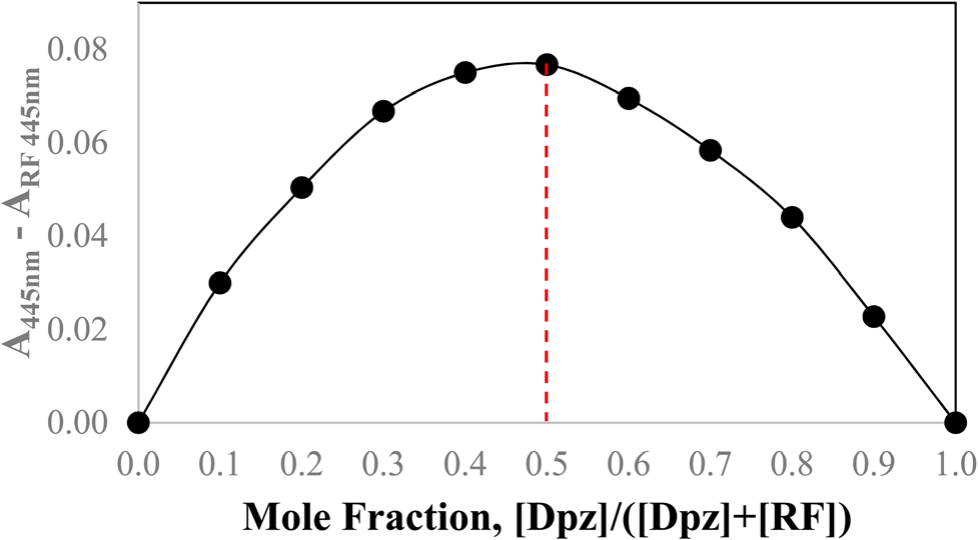
Job's plot for DPZ: RF (1 : 1) complex in an aqueous solution at pH 7.0.

The stoichiometric complexation between DPZ and RF has been evaluated by Job's method of continuous variations. The total concentration of DPZ and RF has been kept constant ([DPZ] + [RF] = 1.00 × 10^−4^ M/100 mL), while the molar fractions have been varied continuously. The change in absorbance (Δ*A*) *versus* molar fraction of DPZ has been plotted and is given in Fig. 8. The Δ*A* value reaches the maximum at a DPZ molar fraction of 0.5, which indicates that there is 1 : 1 complexation between DPZ and RF. Also, it suggests that DPZ interacts with RF at a single binding site. The 1 : 1 binding ratio provides critical insight into the interaction between DPZ and RF, which bind to each other in an equimolar concentration.

### 3.6 Photodegradation kinetics of DPZ alone and in the presence of RF

The photodegradation kinetics studies in the whole pH range (pH 2.0–12.0) are carried out using buffer solutions to maintain the pH of the medium during photolytic reactions. The pH of solutions fluctuates during photolytic reactions and due to drug–solvent interactions, which results in erroneous kinetic data. Drugs are present in their ionized and non-ionized forms in aqueous solutions, depending on the pH of the medium, showing different susceptibilities to light-induced degradation. Therefore, buffer solutions have been used to maintain the pH of the medium to evaluate which form of DPZ (ionized or non-ionized) is more susceptible to photodegradation, to determine the optimum pH for stability, and to mimic the physiological pH. In an aqueous solution (pH 2.0–12.0), the photodegradation of DPZ (1.00 × 10^−4^ M) alone and in the presence of different concentrations of RF (0.05–0.50 × 10^−4^ M) was carried out in aerobic and anaerobic conditions under UV and visible irradiation. The obtained molar concentrations of DPZ in the presence of different concentrations of RF (0.05–0.50 × 10^−4^ M) after UV and visible irradiation in aerobic and anaerobic conditions at pH 2.0–12.0 were plotted against time, and it was found that it does not follow zero-order kinetics for its degradation in the presence of RF. The log concentration of DPZ (M × 10^−4^) *versus* time plots were prepared, and it was found that it follows first-order degradation kinetics in the presence of RF under UV and visible light in both aerobic and anaerobic conditions at pH ranging from 2.0 to 12.0. These plots were used to calculate the first-order (*k*_obs_) constants for the photolysis of DPZ in the presence of RF (0.05–0.50 × 10^−4^ M) under both aerobic and anaerobic conditions with UV and visible irradiation (Table 6 and 7). In the absence of RF, the rate constants (*k*_0_) are given in Table 6 and 7. It has been found that the rate of photodegradation of DPZ increases with an increase in the concentration of RF (0.05–0.50 × 10^−4^ M), indicating that RF catalyzes the photodegradation of DPZ (1.00 × 10^−4^ M) under the studied conditions. The first-order rate constants for the photolytic degradation of DPZ in the presence of RF in aerobic and anaerobic conditions under UV and visible irradiation are in the range of 0.25–11.5 and 0.025–1.12 × 10^−2^ min^−1^, respectively (Table 6 and 7). The rates of photodegradation of DPZ in the presence of RF in aerobic conditions are 10 times higher than those in anaerobic conditions. The higher rate of photodegradation for DPZ in the presence of RF in aerobic conditions is due to the DPZ following two different pathways for its RF-sensitized photodegradation, as reported earlier in the case of ascorbic acid^48,140,146^ and pyridoxine.^49^ The rate of RF-sensitized degradation of DPZ is higher due to the involvement of oxygen in the degradation reactions. The molecular oxygen (^3^O_2_), when it interacts with the excited triplet state of RF, forms singlet oxygen (^1^O_2_), O_2_^−^ (super anion radicals), and OH˙ (hydroxy radicals) that attack the DPZ molecule, which increases its photodegradation. However, in anaerobic conditions, the absence of ^3^O_2_ results in the retardation of reactive oxygen species (ROS) formation, leading to a decrease in the photodegradation of DPZ in the presence of RF. The rates of photodegradation of DPZ in the presence of RF in aerobic conditions is 10 times higher than in anaerobic conditions, due to RF-sensitized photodegradation of DPZ following both type I and type II pathways simultaneously. RF is known to catalyze the degradation of different substrates (*e.g.*, fenamates,^147^ rhodamine,^148^ ascorbic acid,^48^ pyridoxine,^48^ famotidine,^149^*etc.*), indicating that RF acts as a promoter for the degradation of these compounds. The rate of photodegradation of DPZ is dependent on the concentration of RF. Therefore, the photochemical interaction between DPZ and RF has been evaluated by calculating the second-order rate constants (*k*_2_) under both aerobic and anaerobic conditions, using UV and visible irradiation. The second-order rate constants (*k*_2_) (Table 6 and 7) were calculated by plotting the first-order rate constants (*k*_obs_) *versus* different concentrations of RF, and plots are given in Fig.S7 and S8. The *k*_2_ for the photochemical interaction of DPZ and RF ranges from 0.33 to 8.22 and 0.031 to 0.805 M^−1^ min^−1^, in aerobic and anaerobic conditions under UV and visible irradiations, respectively.

**Table 6:** Rates of photolysis of DPZ in the presence of different concentrations of RF under UV and visible irradiation in aerobic conditions, where *k*_obs_ are first-order rate constants for DPZ photolysis in the presence of RF, *k*_0_ are the rate constants in the absence of RF, and *k*_2_ are the second-order rate constants for the photochemical interaction of DPZ and RF

		Visible light				UV light			
pH	RF concentration (M × 10^4^)	*k*_obs_ × 10^2^ (min^−1^) ± SD	*k*_0_ × 10^2^ (min^−1^)	*k*_2_ (M^−1^, min^−1^)	*Φ*	*k*_obs_ × 10^2^ (min^−1^) ±SD	*k*_0_ × 10^2^ (min^−1^)	*k*_2_ (M^−1^, min^−1^)	*Φ*
2.0	0.10	0.52 ± 0.15	0.14	1.45	—	0.21 ± 0.41	0.06	0.61	—
	0.20	0.79 ± 0.22			—	0.33 ± 0.22			—
	0.30	1.22 ± 0.19			—	0.50 ± 0.56			—
	0.40	1.55 ± 0.14			—	0.65 ± 0.51			—
	0.50	1.95 ± 0.25			0.59	0.82 ± 0.48			0.58
3.0	0.10	0.92 ± 0.33	0.36	2.18	—	0.39 ± 0.73	0.14	0.92	—
	0.20	1.38 ± 0.45			—	0.58 ± 0.21			—
	0.30	1.95 ± 0.62			—	0.82 ± 0.45			—
	0.40	2.50 ± 0.51			—	1.05 ± 0.68			—
	0.50	3.11 ± 0.48			0.94	1.30 ± 0.74			0.93
4.0	0.10	0.61 ± 0.22	0.24	1.44	—	0.26 ± 0.95	0.10	0.60	—
	0.20	0.92 ± 0.41			—	0.39 ± 0.41			—
	0.30	1.30 ± 0.63			—	0.55 ± 0.47			—
	0.40	1.71 ± 0.84			—	0.71 ± 0.53			—
	0.50	2.05 ± 0.21			0.62	0.86 ± 0.68			0.61
5.0	0.10	0.25 ± 0.26	0.65	0.77	—	0.10 ± 0.84	0.02	0.32	—
	0.20	0.43 ± 0.17			—	0.18 ± 0.96			—
	0.30	0.61 ± 0.19			—	0.26 ± 0.74			—
	0.40	0.82 ± 0.25			—	0.34 ± 0.63			—
	0.50	1.02 ± 0.77			0.31	0.43 ± 0.41			0.31
6.0	0.10	0.27 ± 0.41	0.78	0.74	—	0.11 ± 0.14	0.04	0.33	—
	0.20	0.45 ± 0.38			—	0.19 ± 0.19			—
	0.30	0.64 ± 0.62			—	0.27 ± 0.22			—
	0.40	0.82 ± 0.74			—	0.35 ± 0.24			—
	0.50	1.03 ± 0.63			0.32	0.44 ± 0.47			0.32
7.0	0.10	0.65 ± 0.45	0.14	1.37	—	0.25 ± 0.45	0.07	0.59	—
	0.20	1.01 ± 0.10			—	0.42 ± 0.22			—
	0.30	1.36 ± 0.28			—	0.57 ± 0.19			—
	0.40	1.67 ± 0.24			—	0.70 ± 0.88			—
	0.50	2.02 ± 0.25			0.61	0.84 ± 0.85			0.60
8.0	0.10	1.02 ± 0.63	0.20	2.93	—	0.43 ± 0.41	0.11	1.23	—
	0.20	1.73 ± 0.47			—	0.73 ± 0.63			—
	0.30	2.52 ± 0.84			—	1.06 ± 0.41			—
	0.40	3.25 ± 0.42			—	1.36 ± 0.35			—
	0.50	3.95 ± 0.73			1.19	1.66 ± 0.63			1.18
9.0	0.10	1.52 ± 0.21	0.29	4.51	—	0.64 ± 0.41	0.16	1.89	—
	0.20	2.55 ± 0.33			—	1.07 ± 0.58			—
	0.30	3.75 ± 0.47			—	1.57 ± 0.63			—
	0.40	4.92 ± 0.82			—	2.07 ± 0.18			—
	0.50	6.02 ± 0.63			1.81	2.53 ± 0.29			1.80
10.0	0.10	2.13 ± 0.51	0.52	6.27	—	0.92 ± 0.31	0.24	2.60	—
	0.20	3.59 ± 0.96			—	1.51 ± 0.45			—
	0.30	5.20 ± 0.41			—	2.18 ± 0.74			—
	0.40	6.85 ± 0.33			—	2.87 ± 0.19			—
	0.50	8.41 ± 0.65			2.53	3.53 ± 0.39			2.52
11.0	0.10	2.95 ± 0.41	1.10	7.25	—	1.23 ± 0.84	0.42	3.04	—
	0.20	4.62 ± 0.48			—	1.93 ± 0.47			—
	0.30	6.53 ± 0.88			—	2.74 ± 0.65			—
	0.40	8.51 ± 0.46			—	3.57 ± 0.74			—
	0.50	10.2 ± 0.51			3.07	4.28 ± 0.36			3.05
12.0	0.10	3.33 ± 0.63	1.11	8.22	—	1.32 ± 0.41	0.46	3.23	—
	0.20	5.12 ± 0.77			—	2.06 ± 0.75			—
	0.30	7.31 ± 0.48			—	2.95 ± 0.19			—
	0.40	9.50 ± 0.92			—	3.85 ± 0.22			—
	0.50	11.5 ± 0.69			3.46	4.55 ± 0.65			3.24

**Table 7:** Rates of photolysis of DPZ in the presence of different concentrations of RF under UV and visible irradiation in anaerobic conditions, where *k*_obs_ are first-order rate constants for DPZ photolysis in the presence of RF, *k*_0_ are the rate constants in the absence of RF, and *k*_2_ are the second-order rate constants for the photochemical interaction of DPZ and RF

		Visible light				UV light			
pH	RF concentration (M × 10^4^)	*k*_obs_ × 10^2^ (min^−1^) ±SD	*k*_0_ × 10^2^ (min^−1^)	*k*_2_ (M^−1^ min^−1^)	*Φ*	*k*_obs_ × 10^2^ (min^−1^) ± SD	*k*_0_ × 10^2^ (min^−1^)	*k*_2_ (M^−1^, min^−1^)	*Φ*
2.0	0.10	0.050 ± 0.25	0.016	0.145	0.059	0.021 ± 0.45	0.005	0.061	0.058
	0.20	0.079 ± 0.16				0.033 ± 0.14			
	0.30	0.120 ± 0.19				0.050 ± 0.63			
	0.40	0.155 ± 0.45				0.065 ± 0.14			
	0.50	0.195 ± 0.63				0.082 ± 0.78			
3.0	0.10	0.092 ± 0.14	0.037	0.218	0.094	0.038 ± 0.63	0.015	0.092	0.093
	0.20	0.138 ± 0.18				0.058 ± 0.15			
	0.30	0.195 ± 0.29				0.082 ± 0.52			
	0.40	0.250 ± 0.33				0.105 ± 0.36			
	0.50	0.311 ± 0.47				0.130 ± 0.47			
4.0	0.10	0.061 ± 0.58	0.025	0.144	0.062	0.026 ± 0.15	0.009	0.060	0.146
	0.20	0.092 ± 0.63				0.039 ± 0.41			
	0.30	0.131 ± 0.14				0.055 ± 0.36			
	0.40	0.170 ± 0.33				0.071 ± 0.48			
	0.50	0.205 ± 0.74				0.086 ± 0.63			
5.0	0.10	0.025 ± 0.14	0.006	0.077	0.031	0.010 ± 0.51	0.002	0.032	0.030
	0.20	0.043 ± 0.28				0.018 ± 0.14			
	0.30	0.061 ± 0.69				0.026 ± 0.33			
	0.40	0.082 ± 0.47				0.034 ± 0.63			
	0.50	0.102 ± 0.36				0.043 ± 0.54			
6.0	0.10	0.027 ± 0.15	0.010	0.074	0.031	0.011 ± 0.59	0.003	0.031	0.031
	0.20	0.045 ± 0.63				0.019 ± 0.41			
	0.30	0.064 ± 0.51				0.027 ± 0.63			
	0.40	0.082 ± 0.84				0.034 ± 0.15			
	0.50	0.102 ± 0.96				0.043 ± 0.95			
7.0	0.10	0.062 ± 0.11	0.021	0.140	0.061	0.025 ± 0.48	0.006	0.059	0.060
	0.20	0.100 ± 0.29				0.042 ± 0.52			
	0.30	0.136 ± 0.22				0.057 ± 0.63			
	0.40	0.167 ± 0.36				0.070 ± 0.41			
	0.50	0.202 ± 0.34				0.085 ± 0.58			
8.0	0.10	0.102 ± 0.51	0.032	0.293	0.119	0.043 ± 0.47	0.012	0.123	0.118
	0.20	0.173 ± 0.85				0.073 ± 0.11			
	0.30	0.252 ± 0.15				0.106 ± 0.22			
	0.40	0.325 ± 0.63				0.136 ± 0.19			
	0.50	0.395 ± 0.74				0.166 ± 0.36			
9.0	0.10	0.152 ± 0.17	0.042	0.450	0.181	0.066 ± 0.41	0.025	0.187	0.178
	0.20	0.255 ± 0.92				0.107 ± 0.25			
	0.30	0.375 ± 0.65				0.157 ± 0.63			
	0.40	0.492 ± 0.45				0.207 ± 0.22			
	0.50	0.602 ± 0.63				0.253 ± 0.41			
10.0	0.10	0.213 ± 0.84	0.059	0.627	0.253	0.095 ± 0.33	0.036	0.258	0.252
	0.20	0.359 ± 0.33				0.151 ± 0.52			
	0.30	0.520 ± 0.14				0.218 ± 0.51			
	0.40	0.685 ± 0.41				0.287 ± 0.22			
	0.50	0.840 ± 0.86				0.353 ± 0.63			
11.0	0.10	0.295 ± 0.41	0.095	0.725	0.307	0.120 ± 0.15	0.045	0.308	0.305
	0.20	0.460 ± 0.63				0.193 ± 0.44			
	0.30	0.653 ± 0.47				0.274 ± 0.63			
	0.40	0.850 ± 0.45				0.357 ± 0.85			
	0.50	1.020 ± 0.33				0.428 ± 0.92			
12.0	0.10	0.315 ± 0.36	0.102	0.805	0.337	0.130 ± 0.49	0.048	0.321	0.322
	0.20	0.499 ± 0.14				0.205 ± 0.85			
	0.30	0.715 ± 0.69				0.289 ± 0.75			
	0.40	0.920 ± 0.36				0.376 ± 0.69			
	0.50	1.120 ± 0.59				0.452 ± 0.88			

### 3.7 Photolysis quantum yields (*Φ*)

The photolysis quantum yields (*Φ*) depend on the reactivity of the excited triplet state in photodegradation reactions. It has been found that *Φ* is higher in aerobic conditions than in anaerobic conditions under visible and UV irradiation. The values of *Φ* range from 0.58–3.46 to 0.058–0.337 (Table 6 and 7) in aerobic and anaerobic conditions, respectively. These higher and lower values of *Φ* indicate the high and low susceptibility of the excited triplet state to the photolysis reactions, respectively.

### 3.8 *k*-pH profile

**Fig. 9:**
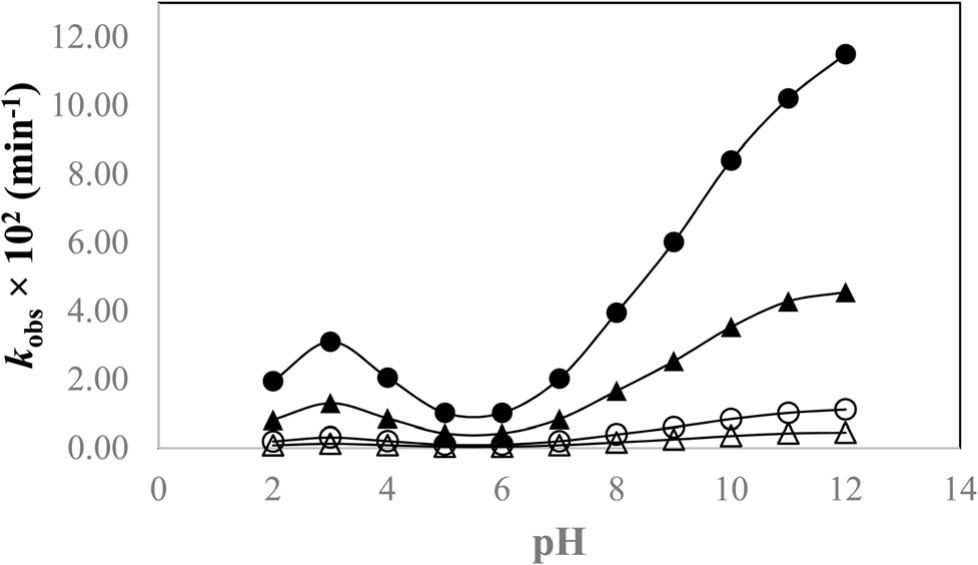
k_obs_-pH profile for the photolysis of DPZ (1.00 × 10^−4^ M) in the presence of RF (0.50 × 10^−4^ M). Where (●, ▲) are for visible and UV irradiations in aerobic conditions, and (○, Δ) are for visible and UV irradiations in anaerobic conditions.

Stability studies are necessary to assess the stability of liquid or co-formulations containing two or more therapeutic agents. Their stability can be altered due to any photochemical interaction and reactions involving photosensitizers. The evaluation of the photochemical interaction between DPZ and RF was conducted by plotting the rate constants against pH (Fig. 9) under both aerobic and anaerobic conditions, using UV and visible irradiation. The *k*-pH profile shows a bell-shaped curve from pH 2.0 to 4.0 and a sigmoid curve from pH 5.0 to 12.0. The rate of photodegradation of DPZ in the presence of RF increases in aerobic and anaerobic conditions. DPZ, when dissolved in aqueous solution, exists in cationic (<pH 9.0) and neutral species (>pH 9.0). The tertiary amine group in the piperidine ring accepts a proton below pH 9.0, resulting in the formation of the cationic form. Whereas, at pH greater than 9.0, the tertiary amine groups remain unprotonated to form neutral species of DPZ. However, DPZ does not exist in anionic form.^150^ RF acts as a promoter (photosensitizer) in the photodegradation of DPZ, and it interacts with ionic species (cationic and neutral) differently, resulting in an enhanced or slow photodegradation of DPZ. It reacts with the ^3^O_2_ to form reactive oxygen species (ROS), which interact with the different ionic species of DPZ (cationic, neutral) and thereby affect the rates of photodegradation of DPZ.

The rate of photodegradation of DPZ in the presence of RF in aerobic and anaerobic conditions under UV and visible irradiation increases from pH 2.0 to 3.0, where the rate is maximum (pH 3.0). After a pH of 3.0, the rate of photodegradation decreases to a pH of 5.0, forming a bell-shaped curve. At pH 3.0, the DPZ exists in its 99.99% cationic form, which is highly susceptible to interacting with ^1^O_2_, resulting in a higher rate of photodegradation of DPZ at this pH. After pH 3.0, the rate of degradation decreases until pH 5.0, at which point the rate of photodegradation of DPZ is at a minimum. However, after a pH of 5.0, the rate of photodegradation increases to a pH of 12.0, forming a sigmoid curve. After pH 8.0 (*pK*_a_ 8.95,^151,152^ there is a sharp increase in the rate of photodegradation of DPZ in aerobic and anaerobic conditions. At pH 12.0, DPZ exists in its neutral form (99.99%), which is highly susceptible to interacting with ROS, resulting in the increased photodegradation rates of DPZ in the presence of RF.

### 3.9 Computational analysis

#### 3.9.1 Frontier molecular orbital (FMO) analysis

**Fig. 10:**
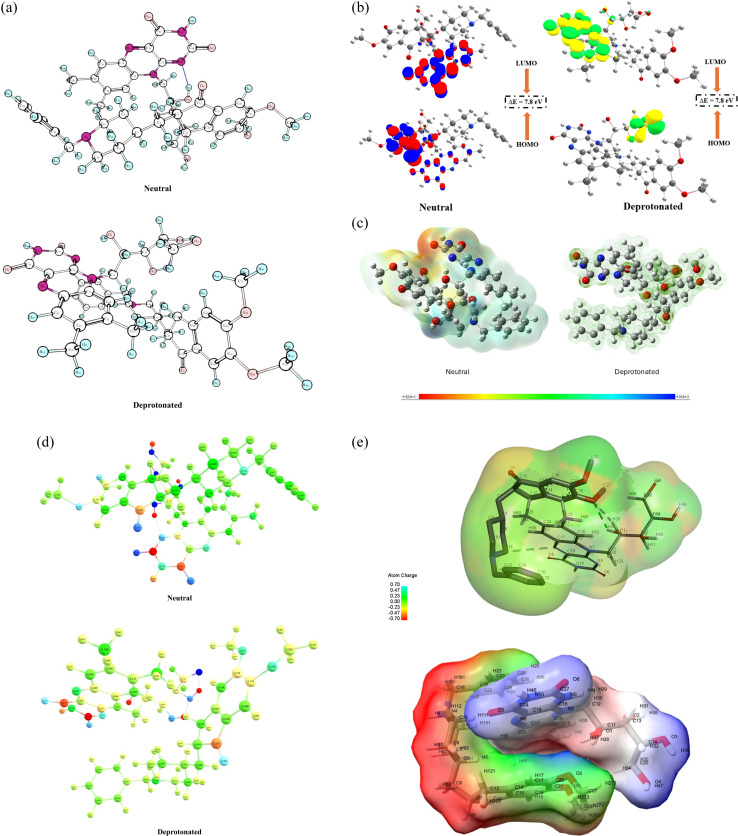
(a) Optimized structure of neutral and deprotonated complex.

The electronic characteristics and reactivity of compounds are understood by the FMO analysis, which has gained significant importance. The molecular orbitals provide information about the electronic and optical properties, photochemical reactions, quantum chemistry, and biological mechanisms. Although DFT methods such as B3LYP/6-31G(d) are known to provide more accurate HOMO–LUMO energy gaps, the HF/6-31G(d) level was employed in this study as a computationally efficient alternative due to system size and hardware limitations. The method was sufficient for obtaining reliable optimized geometries and qualitative orbital descriptions.^153–155^ The optimized structures of neutral and deprotonated forms of the complex are shown in Fig. 10a. The electron transfer occurs mainly from the ground state to the excited state in the frontier molecular orbitals. The kinetic stability and reactivity of the compounds can also be explained from the energy difference of HOMO and LUMO (Δ*E*). Kinetically, a high Δ*E* suggests good stability and less reactivity of a compound. The FMO for complexes of DPZ-RF in neutral and deprotonated states is shown in Fig. 10b. The calculated energies for the value of HOMO and LUMO are −0.309 and 0.0176 Hartree, respectively. The value of Δ*E* (Δ*E*_HOMO–LUMO_) for the neutral complex was found to be 7.8 eV. suggesting it to be more stable and less reactive after complex formation. The visual representation of frontier orbitals showed localization of LUMO on RF, while HOMO on DPZ. A large energy barrier of 7.8 eV suggests a lower likelihood of photoinduced charge transfer, causing a reduced RF-induced photolysis. This could be possible due to delocalization of charge density, alteration of orbital energies, or prevention of direct energy transfer from excited RF to DPZ. The FMO analysis of the deprotonated form of the complex was also studied using the same method to understand the behavior of electron transfer in alkaline pH. The energy gap is 2.176 eV in the protonated form of the complex. This low energy difference suggests the complex is less stable and more reactive in alkaline pH. The frontier orbital visualization also showed localization of HOMO and LUMO orbitals in the RF molecule. This could suggest that RF is the primary electron donor in the complex. The aromatic ring of RF holds the highest energy orbitals and is involved in charge transfer or photochemical reactions, however, the side chain of RF, that is the ribityl moiety, might act as an electron acceptor, indicating intramolecular charge transfer within RF. DPZ, on the other hand, shows no direct involvement in the frontier orbital interactions of the complex. It might be possible that DPZ has a structural or steric role rather than photochemical. This could also suggest the dominating role of RF in electronic structure, while in deprotonated form, potentially influencing DPZ photodegradation through indirect non-covalent interactions such as hydrogen bonding or π–π stacking. The Mulliken charge and molecular ligand–ligand interaction would further assist in understanding the type of interactions involved between RF and DPZ.

Although our FMO results for the neutral complex suggest a large HOMO–LUMO gap and ground-state stability, literature precedent shows that RF becomes more photochemically reactive when deprotonated. For example, studies demonstrate enhanced degradation of RF under alkaline pH (*i.e.*, when it forms its anionic species) under light irradiation.^156–158^ In these cases, photoexcitation leads to electron transfer or radical formation even for systems that appear stable in the ground state. Thus, the observed experimental degradation likely proceeds through excited-state mechanisms that are promoted under high pH conditions, which is consistent with our hypothesis regarding deprotonation-induced photolysis (Fig. 9).

#### 3.9.2 Molecular electrostatic potential (MEP)

The MEP can help in displaying the electron density to find the positions of nucleophilic and electrophilic reactions and hydrogen bonding.^159^ MEP provides a 3D graph to study the charge distribution and properties related to the charge of the molecules. A relative polarity of the molecule can also be studied with the help of this graph. The regions that are avoided by electrophiles or nucleophiles can also be detected through MEP. A chemical system tends to create an electrostatic potential around itself that causes it to experience attractive or repulsive forces in a region by using a hypothetical “volumeness” unit of positive charge as a probe, causing the electrostatic potential to be negative or positive, respectively. The electrophilic reactivity, which is electron-rich sites are depicted in red color, while the nucleophilic reactivity, which is electron-deficient sites, is shown in blue color in the MEP maps. The MEP maps for the neutral and deprotonated DPZ-RF complexes are given in Fig. 10c. In the neutral complex, the negative potential zones (red) localized over the carbonyl and N(10)–C(4a) regions of RF indicate potential hydrogen-bond acceptor sites, whereas the positive potential regions (blue) on the tertiary amine and aromatic benzyl ring of DPZ suggest proton-accepting and π-stacking donor regions. This complementary charge distribution supports the likelihood of non-covalent stabilization through hydrogen bonding and π–π interactions. In contrast, the deprotonated complex shows a pronounced shift of negative potential across the RF isoalloxazine ring, suggesting enhanced electron-donating ability. Such redistribution may facilitate photoinduced electron transfer, leading to the generation of ROS and the subsequent oxidative degradation of DPZ at its electron-deficient sites.

#### 3.9.3 Mulliken charge analysis

The Mulliken charge analysis describes the electronic distribution in a molecule based on quantum chemical calculations.^160^ The Mulliken atomic charges were calculated for the neutral state of the complex to investigate the electronic structure and multipole moments of the neutral complex using hf6-31g(d) method to estimate atomic charges and electron density distribution (Fig. 10d). The electronic spatial extent (<*R*^2^> = 38605.91A.U.) shows a highly delocalized electronic cloud consistent with an extended π-conjugated system. The higher-order moments, such as quadrupole, octapole, and hexapole, further support the anisotropic distribution of electron density within the molecular framework. In the deprotonated complex, the total charge of −1.00 confirms the deprotonated species. A significant localization of negative charge on oxygen atoms, particularly, O4(−0.797), suggested the deprotonated site of the complex. The overall negative charge and the large dipole moment (11.50D) confirm the polar and reactive nature of the complex. A significant reorientation of the dipole vector in the deprotonated state was observed with the dipole predominantly aligned along the *z*-axis, whereas in the neutral form, it spread across all three axes. This suggests redistribution of electronic density following proton removal. The difference in Mulliken charges between the neutral and deprotonated states was also compared to understand the redistribution of charges after deprotonation, as shown in Table S7. It was observed that the neighboring oxygen and nitrogen atoms, along with carbon in the RF ring, showed ΔCharge(_Deprot–Neutral_) > 0.3, suggesting a significant change in electronic distribution. A more positive charge was also observed after deprotonation of the complex in the DPZ moiety, suggesting it to be a better electron acceptor and probable susceptibility towards photoinduced electron transfer, which leads to the RF sensitized photodegradation of DPZ.

#### 3.9.4 Molecular docking analysis

In the docking analysis, DPZ was treated as the receptor molecule and RF as the ligand. A blind docking grid was defined around the entire DPZ surface in PyRx to allow full exploration of potential interaction sites. This approach, while less common than protein–ligand docking, has been successfully applied to study ligand–ligand and host–guest interactions. The map of charge distribution was created through soft interpolated surface visualization in BIOVIA Discovery Studio after performing ligand–ligand docking using PyRx, which indicated clear electron-rich (red) and electron-deficient (blue) areas on the ligand. Notably, red patches potentially act as hydrogen bond acceptors, indicating electron accumulation sites, while blue zones indicate donor interactions, suggesting electrophilic centers. This charge distribution plays a critical role in stabilizing the ligand (DPZ) binding with the host (RF) molecule under neutral conditions. The ligand–ligand interaction of DPZ and RF under neutral conditions is given in Fig. 10d, and it was found that potential hydrogen bonding and π–π stacking are prominent.

### 3.10 TOC analysis

TOC analysis has been used to evaluate the mineralization of DPZ, RF, and DPZ–RF. The results obtained after TOC analysis are presented in Table 8, indicating that the undegraded DPZ, RF, and DPZ-RF yield higher TOC values. These TOC values reduce after photolytic degradation, which represents the mineralization and high photodegradation as reported earlier.^49,161,162^ These TOC values significantly decrease, indicating the degradation of DPZ, RF, and DPZ-RF, which results in the formation of smaller fragments, CO_2_, and inorganic substances, which subsequently lower the TOC content in the photolyzed solutions. The TOC loss after irradiation of DPZ, RF, and DPZ-RF is 72.33, 92.39, and 40.0%, respectively (Table 8). The greater loss of TOC is in RF alone (92.3%), followed by DPZ (72.3%) and DPZ-RF (40%). The percentage loss of TOC does not depend on how fast the parent molecule will be consumed, but also depends on the mineralization of its degradation products. RF is known to photooxidize to form CMF, which subsequently degrades into ring cleavage products^163^ that are readily mineralized, resulting in the higher percent loss of TOC. However, in the DPZ-RF mixture, the lower loss of TOC may be due to the formation of stable nitrogen-containing products (*i.e.*, quaternary ammonium fragments and azo/imine species) by energy transfer or electron transfer from RF to DPZ that inhibit complete oxidation. Also, the formation of ground or excited state complexes of DPZ-RF when it degrades results in the formation of large molecules as degradation products, which are less oxidizable, resulting in reduced mineralization and decreased TOC loss, as evident from the results obtained from the TOC analysis.

**Table 8:** Analysis of TOC for DPZ (1.00 × 10^−4^ M), RF (0.50 × 10^−4^ M). and DPZ-RF at pH 7.0 before and after photolysis using visible irradiation

	Before irradiation			After irradiation			Total TOC loss (%)
	TOC^a^ (ppb) ± SD^d^	IC^b^ (ppb) ± SD^d^	TC^c^ (ppb) ± SD	TOC^a^ (ppb) ± SD^d^	IC^b^ (ppb) ± SD^d^	TC^c^ (ppb) ± SD	
DPZ	228 ± 0.22	697 ± 0.15	925 ± 0.41	63.1 ± 0.47	821.9 ± 0.44	885 ± 0.11	72.33
RF	323 ± 0.36	692 ± 0.47	1015 ± 0.56	24.6 ± 0.52	775.4 ± 0.74	800 ± 0.36	92.39
DPZ-RF	696 ± 0.63	924 ± 0.51	1620 ± 0.74	421.0 ± 0.74	699.0 ± 0.82	1120 ± 0.88	40.00

a Total organic carbon.

b Inorganic carbon.

c Total carbon.

d *n* = 3.

### 3.11 Mode of photodegradation

The photodegradation of DPZ, alone and in the presence of RF, has been carried out under both aerobic and anaerobic conditions. The rates of photodegradation of DPZ are higher in aerobic conditions than in anaerobic conditions. These rates of photodegradation in aerobic conditions are higher due to two separate pathways (type I and type II) followed for their degradation in the presence of RF.^164^ The modes of photodegradation of DPZ, both alone and in the presence of RF, under aerobic and anaerobic conditions, are discussed in the following sections.

#### 3.11.1 DPZ photodegradation in aqueous solution

DPZ, when it absorbs light, converts it into the excited singlet state (^1^[DPZ]) (2). This ^1^[DPZ] undergoes intersystem crossing (isc) and is converted into the excited triplet state (^3^[DPZ]) (3), which further interacts with the ground state (^0^[DPZ]), leading to the formation of the cationic form (DPZ˙^+^) (4). This DPZ˙^+^ loses an H^+^ atom to form the oxidized form of DPZ (DPZ˙) (5), which is further oxidized to form the degradation products of DPZ (6).2
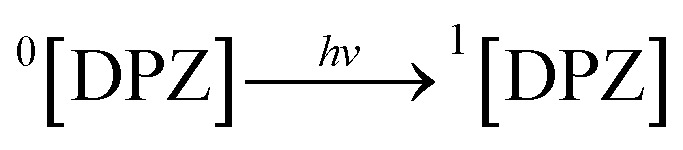
3
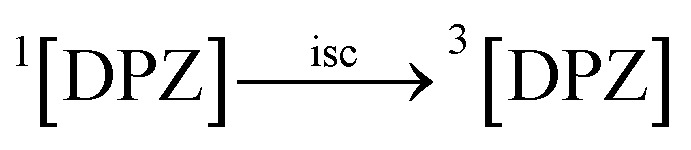

4 ^3^[DPZ] + ^1^[DPZ] → DPZ˙^+^

5
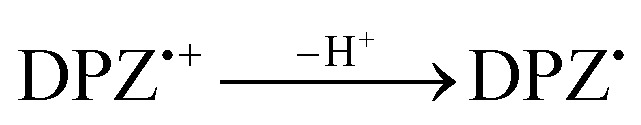
6
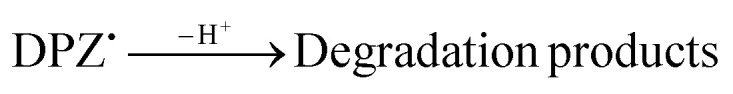


#### 3.11.2 Photochemical interaction of DPZ and RF

RF is known as a potent photosensitizer, resulting in the photosensitized degradation of substrates *via* type I and type II oxidation.^48,49,164,165^ The type I oxidation pathway occurs by the formation of radicals due to the transfer of a proton or an electron in the excited state of RF and substrates. However, in the type II pathway, the ^3^O_2_ is converted into ^1^O_2_ through energy transfer, which results in the oxidation of substrates. It has been found that RF enhances the rate of photodegradation of DPZ in aerobic and anaerobic conditions. The photodegradation mechanisms of DPZ in the presence of RF in aerobic and anaerobic conditions are discussed below.

##### 3.11.2.1 Aerobic conditions

###### 3.11.2.1.1 Type I mechanism

RF, when absorbed, light is converted into the excited singlet state (^1^[RF]) (7), which *via* isc is converted into the excited triplet state (^3^[RF]) (8). This ^3^[RF] interacts with DPZ to form a cationic DPZ radical (DPZ˙^+^) and anionic RF radical (RF˙^−^) (9). The DPZ˙^+^ loses a proton (−H^+^) to form the DPZ radical (DPZ^.^) (10). DPZ^.^ interacts with oxygen to form degradation products and H_2_O_2_ (11).7
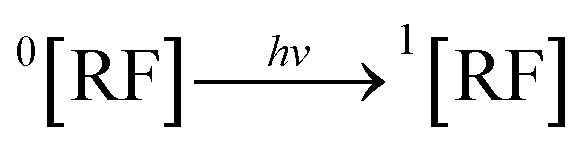
8
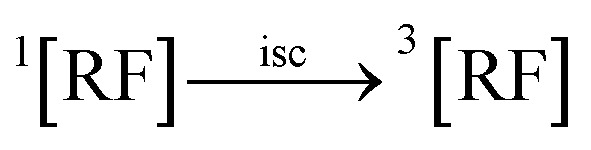

9 ^3^[RF] + DPZ → RF˙^−^ + DPZ˙^+^

10
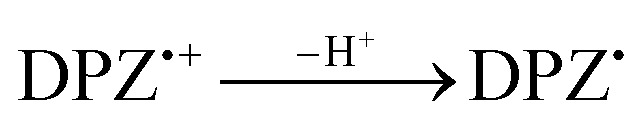

11 DPZ˙ + O_2_ → Degradation products + H_2_O_2_

###### 3.11.2.1.2 Type II mechanism

RF *via* absorbing light is converted into ^1^[RF] (12), which is subsequently transferred through isc into ^3^[RF] (13). This ^3^[RF] reacts with ^3^O_2_ to form ^1^O_2_*via* energy transfer from ^3^[RF] and ground state RF (^0^[RF]) (14). The cationic form of DPZ reacts with ^1^O_2_ and a proton (H^+^) from the aqueous medium to form degradation products of DPZ and H_2_O_2_ (15).12
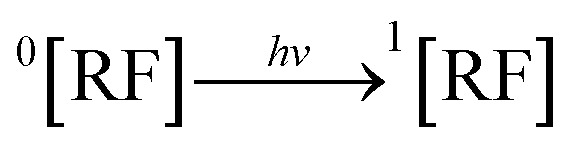
13
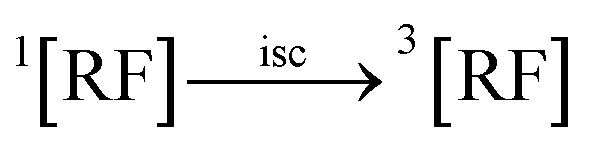

14 ^3^[RF] + ^3^O_2_ → ^0^[RF] + ^1^O_2_

15 DPZ^+^ + ^1^O_2_ + H^+^ → Degradation products + H_2_O_2_

##### 3.11.2.2 Anaerobic conditions

The ground state RF (^0^[RF]) interacts with DPZ^+^ to form a ground state complex (^0^[RF…DPZ^+^]) (16) as evident from the computational analysis (Section 3.9.4, Fig. 10d). This ^0^[RF…DPZ^+^] absorbed light to form the excited singlet state complex (^1^[RF…DPZ^+^]) (17), which is *via* isc converted into the exciplex/excited triplet state complex (^3^[RF…DPZ^+^]) (18).^166^ This ^3^[RF…DPZ^+^] undergoes H-abstraction to form semiquinone radical (RFH˙) and degradation products of DPZ (19). The two semiquinone radicals (2RFH˙), through disproportionation, form oxidized RF (RF_OX_) and reduced RF (RFH_2_, dihydroriboflavin) (20). The RFH_2_ reacts with ^3^O_2_ to form cationic RFH_2_˙^+^ (cationic RF radical) and O_2_^.–^ (anionic superoxide radical) (21). These RFH_2_˙^+^ and O_2_˙^−^ radicals recombine *via* radical–radical recombination, leading to the formation of RF and H_2_O_2_ (22). The ^3^[RF] interact with ^3^O_2_ to form ground state RF (^0^[RF]) and ^1^O_2_ (23) and which is converted into ^3^O_2_ (24).

16 ^0^[RF] + DPZ^+^ → ^0^[RF…DPZ^+^]

17
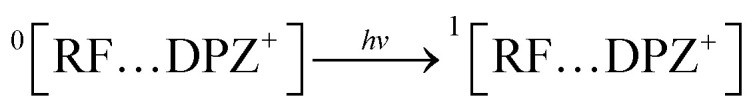
18
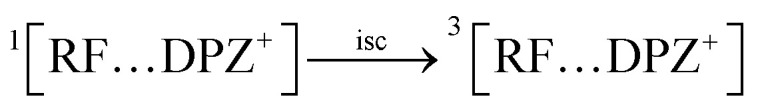
19
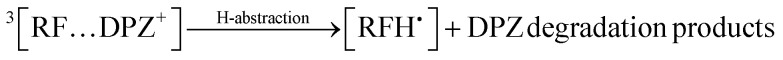
20
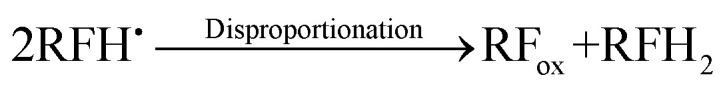
21
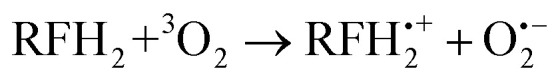
22
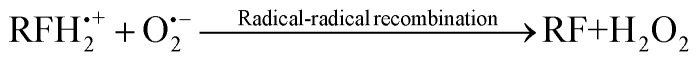

23 ^3^[RF] + ^3^O_2_ → ^0^[RF] + ^1^O_2_

24 ^1^O_2_ → ^3^O_2_

## 4 Conclusions

The present study aims to provide pharmaceutical formulators with an idea for developing a stable co-formulation of donepezil (DPZ) and riboflavin (RF) to address the needs of elderly patients. The photodegradation of DPZ increases with an increase in RF concentration, indicating that RF acts as an enhancer for the photodegradation of DPZ. The rate of degradation forms a bell-shaped curve from pH 2.0 to 4.0 and from pH 5.0 to 12.0, forming a sigmoid curve, indicating that these two can be formulated at pH 5.0, where there is a minimum rate of photolysis of DPZ in the presence of RF. The rate of photodegradation of DPZ is maximum at pH 3.0 and 12.0, where the cationic and neutral species are approximately 99.99%, indicating that these two forms are highly susceptible to photodegradation in the presence of RF. Two green analytical methods, two-component spectrometric and HPLC, have been developed and validated for the simultaneous determination of DPZ and RF. It was found that both methods are equally effective for quantitative estimation of DPZ and RF in pure and degraded solutions. However, from the green chemistry perspective, the two-component spectrometric method is better than the HPLC method because of its lower energy consumption, use of non-toxic solvents, and good practical applicability.

## Author contributions

TK, ZA: methodology, concept, and design. RA, ZA: writing of the original manuscript. TK, AA, AN, MU, MAE, AK, TA: collection of data. ZA, SA, MAS: reveiweing of the manuscript. ZA: supervision.

## Conflicts of interest

The authors declare that there is no conflict of interest.

## Supplementary Material

RA-015-D5RA07553J-s001

## Data Availability

The data that support the findings of this study are available in the supplementary information (SI). Supplementary information is available. See DOI: https://doi.org/10.1039/d5ra07553j.
