# Supplementary material for: Riboflavin-sensitized photoinduced degradation of donepezil hydrochloride: kinetic and computational insights for pharmaceutical formulation
Source: RSC Adv. 2025 Nov 25;15(53):45799–821. doi: 10.1039/d5ra07553j (PMC12645192; doi:10.1039/d5ra07553j)
Supplement: RA-015-D5RA07553J-s001 [file RA-015-D5RA07553J-s001.pdf]

Supplementary Information (SI) for RSC Advances.
This journal is © The Royal Society of Chemistry 2025

SUPPLEMENTARY DATA

T. Khan et al

Research Article

Riboflavin-Sensitized Photoinduced Degradation of Donepezil Hydrochloride: Kinetic and Computational Insights for Pharmaceutical Formulation

Tooba Khan[1†], Zubair Anwar[1†*], Reem Altaf[2*], Ayesha Awan[3], Aisha Noreen[1], Muneeba Usmani[4], Muhammad Ahsan Ejaz[1], Anam Khan[4,5], Sofia Ahmed[4], Muhammad Ali Sheraz[4], Toufeeque Ali[6]

[1]Department of Pharmaceutical Chemistry, Baqai Institute of Pharmaceutical Sciences, Baqai Medical University, Gadap Road, Superhighway, Karachi–75340, Pakistan

[2] Department of Pharmaceutical Chemistry, Faculty of Pharmacy, Capital University of Science and Technology, Islamabad Expressway, Kahuta Road, Zone-V, Islamabad.

[3]Department of Pharmacognosy, Faculty of Pharmaceutical Sciences, Riphah International University, G-7/4, 7th Avenue, Islamabad

[4]Department of Pharmaceutics, Baqai Institute of Pharmaceutical Sciences, Baqai Medical University, Gadap Road, Superhighway, Karachi–75340, Pakistan

[5]Indus Pharma Pvt. Ltd. Plot 26,27 & 65 Sector 27, Korangi Industrial Area, Karachi, Pakistan.

[6]Liaquat University Hospital, Jamshoro, Pakistan

[†] Shared First Authors

[*] Corresponding author: zubair_ana@hotmail.com / zubair_ana@baqai.edu.pk / reem@cust.edu.pk

Zubair Anwar: 0000-0003-3822-7588
Sofia Ahmed: 0000-0002-0731-1237
Muhammad Ali Sheraz: 0000-0002-6333-8178

Purity Confirmation

1.1 FTIR Spectroscopy

The purity of DPZ and RF was determined using the Nicolet iS5 FTIR spectrophotometer with ZnSE0 windows (Thermo Fisher Scientific, USA). The DPZ and RF samples were placed on a diamond ATR crystal (iD7ATR, ThermoFisher Scientific, Great Britain) to collect spectra after performing 156 scans in the 4000 to 700 cm^{-1} range. The spectra obtained were analyzed using built-in Omnic software (Version 9.0).

1.2 Differential Scanning Calorimetry (DSC)

The DPZ and RF purity was further confirmed by differential scanning calorimetry (Model DSC 100, lab kits, Hong Kong). The DSC instrument was calibrated using the standard indium and zinc. DPZ and RF were weighed (5.0 ± 0.5 mg) in the pans of aluminum, and heating was carried out at 10 °C min^{-1}, under a nitrogen flow (20 ml min^{-1}) between temperatures 30 and 400 °C.

2. Assay Method Validation

2.1 System suitability

It was performed by injecting six replicates of the standard solutions of DPZ (1.00 $\times 10^{-4}$ M), and RF (0.50 $\times 10^{-4}$ M) before analyzing the samples to determine the retention time (t_R), theoretical plates (N), and tailing factor (T).

2.2 Linearity and Range

The linearity of the developed spectrometric and HPLC assay methods for analyzing RF and DPZ was assessed in the concentration ranges of 0.1-1.0 and 0.05-0.5 $\times$ 10^{-4} M, respectively. The calibration curve was prepared by plotting the absorbance and peak area (A.U.) versus the respective concentrations of DPZ and RF. The statistical calculations were carried out on the results

obtained to estimate the correlation coefficient, slope, intercept, standard deviation of the intercept, and standard error of the slope and intercept. The range selection was optimized based on the results obtained from the linearity study.

2.3 Accuracy

Three different concentrations of DPZ (0.1, 0.3, 0.5 × 10^{-4} M) and RF (0.1, 0.3, 0.5 × 10^{-4} M) were selected based on the results obtained from the linearity and range studies. Each measurement was carried out in triplicate, and the percentage recoveries were calculated using the following equation.

$$\text{Percent recovery (\%)} = \frac{\text{Amount Added}}{\text{Amount Found}} \times 100 \qquad (1.0)$$

where,

$$\text{Amount Found} = \frac{\text{Mean of 3 determinations - Intercept}}{\text{Slope}} \qquad (2.0)$$

The relative accuracy error (%) was also calculated using:

$$\text{Relative accuracy error (\%)} = \frac{\text{Recovery (\%) - Mean recovery (\%)}}{\text{Mean recovery (\%)}} \times 100 \qquad (3.0)$$

2.4 Precision

The precision (repeatability and intermediate) of the developed method was estimated by preparing six individual concentrations of DPZ (0.70 × 10^{-4} M) and PD (0.40 × 10^{-4} M), which were subjected

to spectrometric and HPLC analysis. The relative standard deviation (%) was calculated using the following formula to determine precision.

$$\text{RSD (\%)} = \frac{\text{SD}}{\text{Mean}} \times 100 \tag{4.0}$$

2.5 Sensitivity

The sensitivity of the proposed methods is defined by the minimum quantity of analyte that can be detected (LOD) and quantified (LOQ). Linearity data is used to calculate the sensitivity of the proposed methods by following the formula.

$$\text{LOD} = 3.3 \times \frac{\sigma}{S} \tag{5.0}$$

$$\text{LOD} = 10 \times \frac{\sigma}{S} \tag{6.0}$$

where σ is the standard deviation of the y-intercept and S is the slope of the calibration curve.

2.6 Robustness

Deliberate changes were made to the conditions of the proposed methods to assess their robustness. These changes include variations in pH (±0.1 units), detection wavelength (±2 nm), buffer concentration (±0.001 M), and flow rate (±0.1 mL/min).

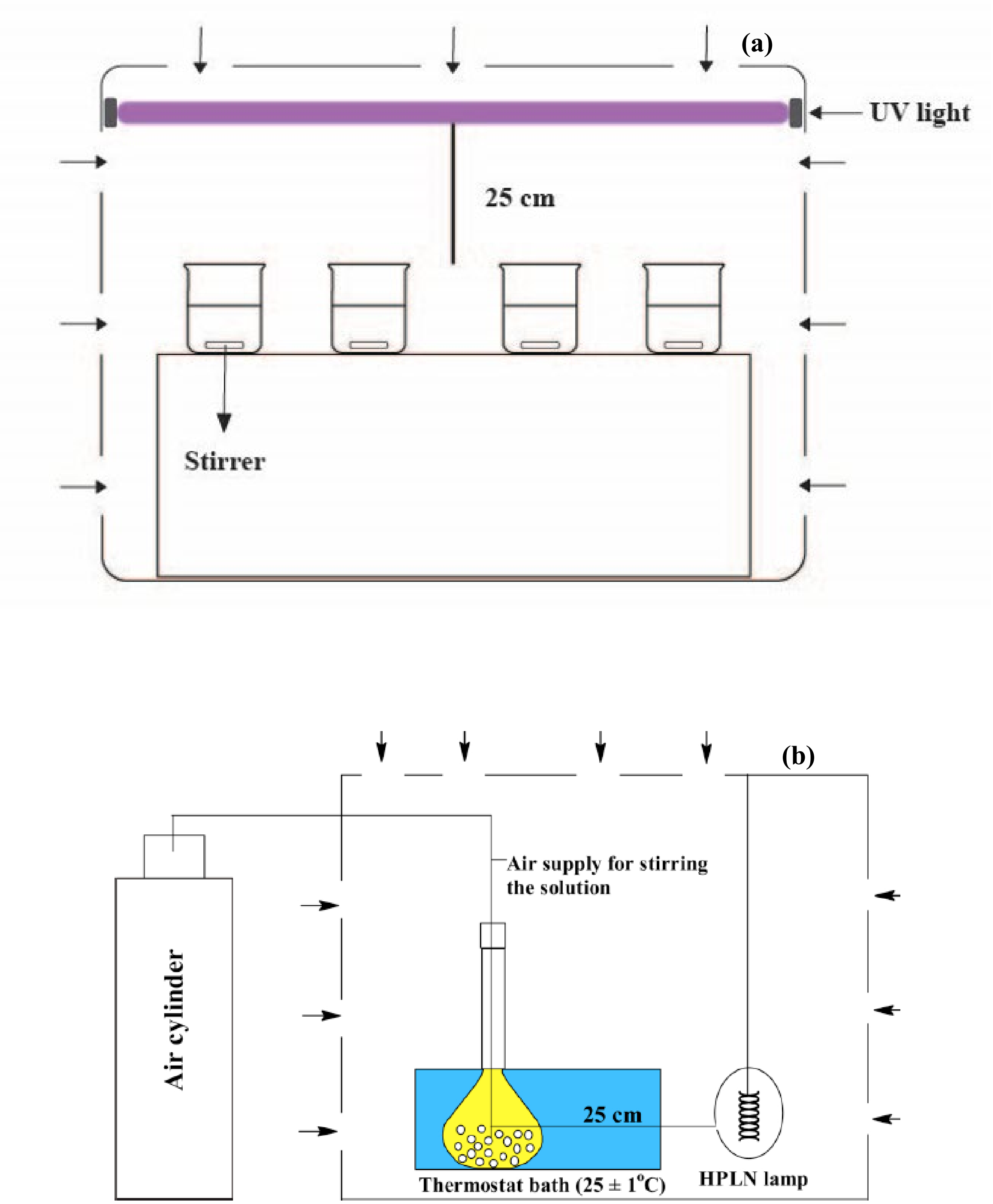

Fig. S1. Irradiation setup for the photolysis of DPZ in the UV (a) and visible (b) irradiation chambers

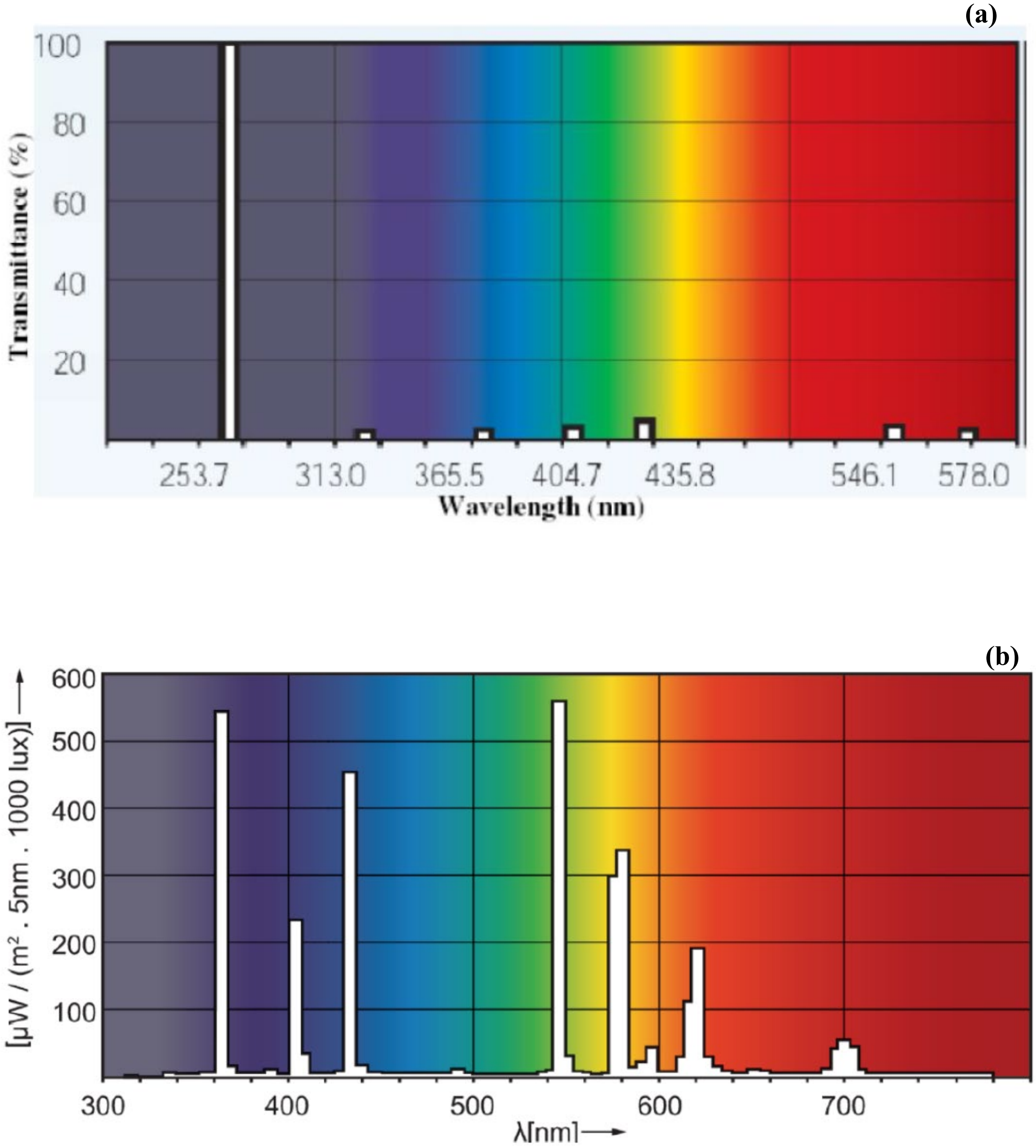

Fig. S2. Spectral emission of UV (a) and visible lamps (b)

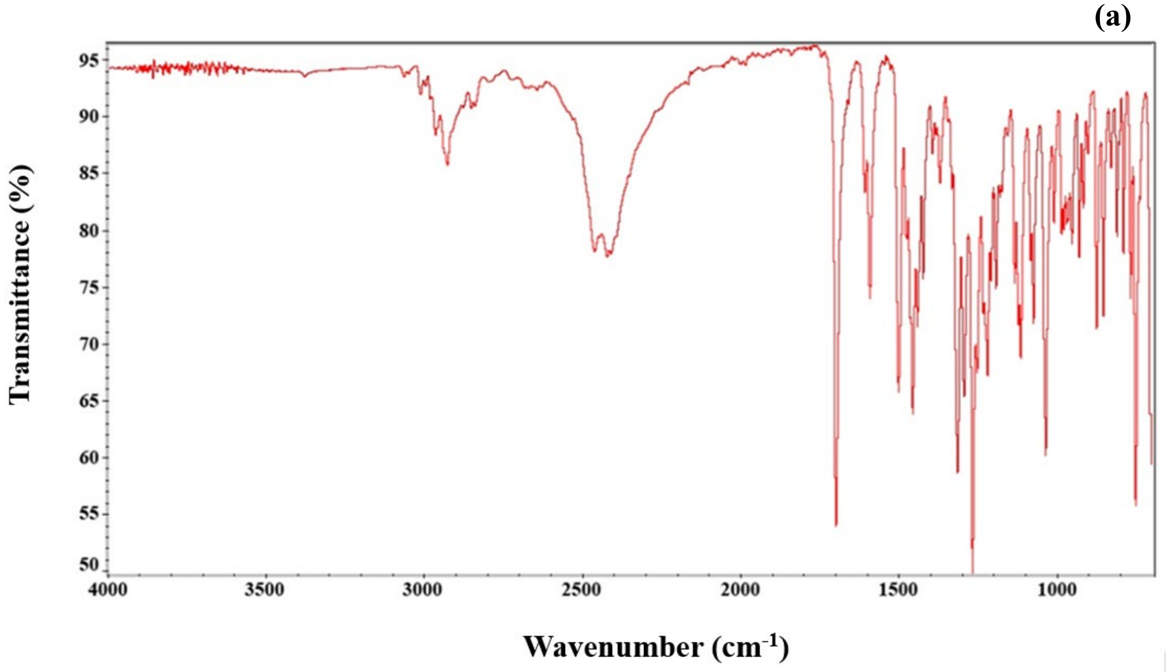

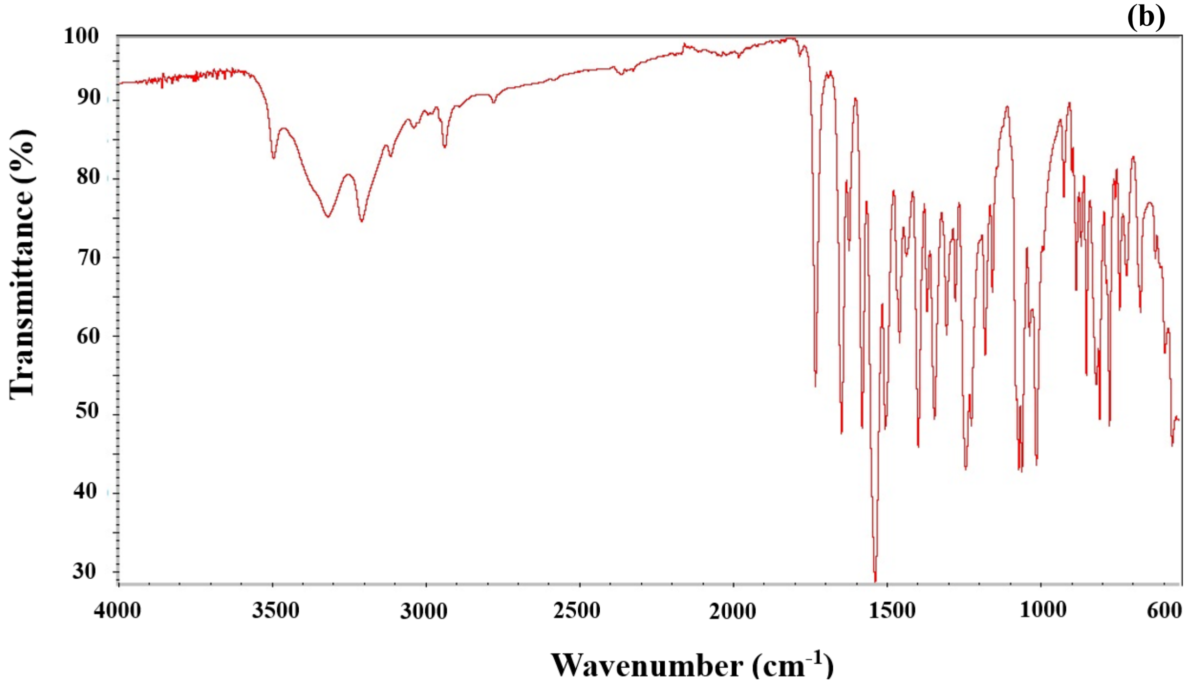

Fig. S3. FTIR spectra of DPZ (a) and RF (b)

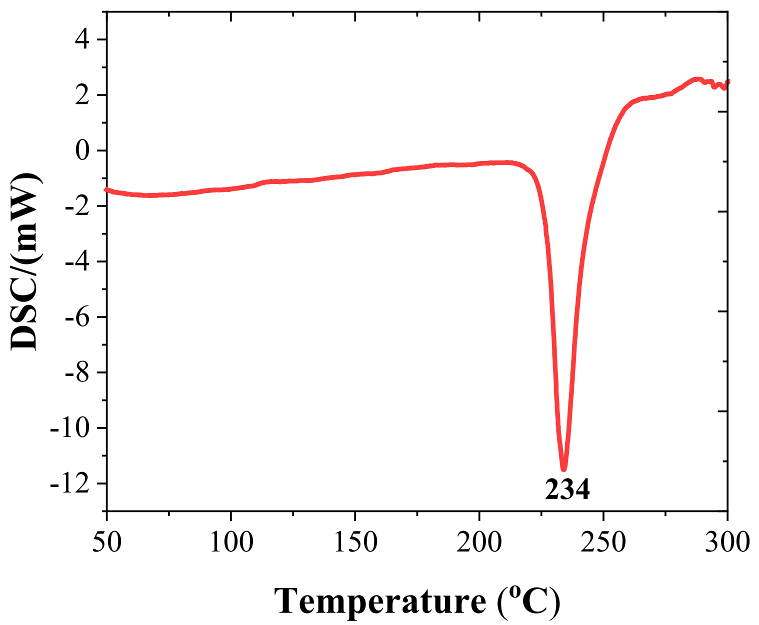

Fig. S4. DSC thermogram of DPZ

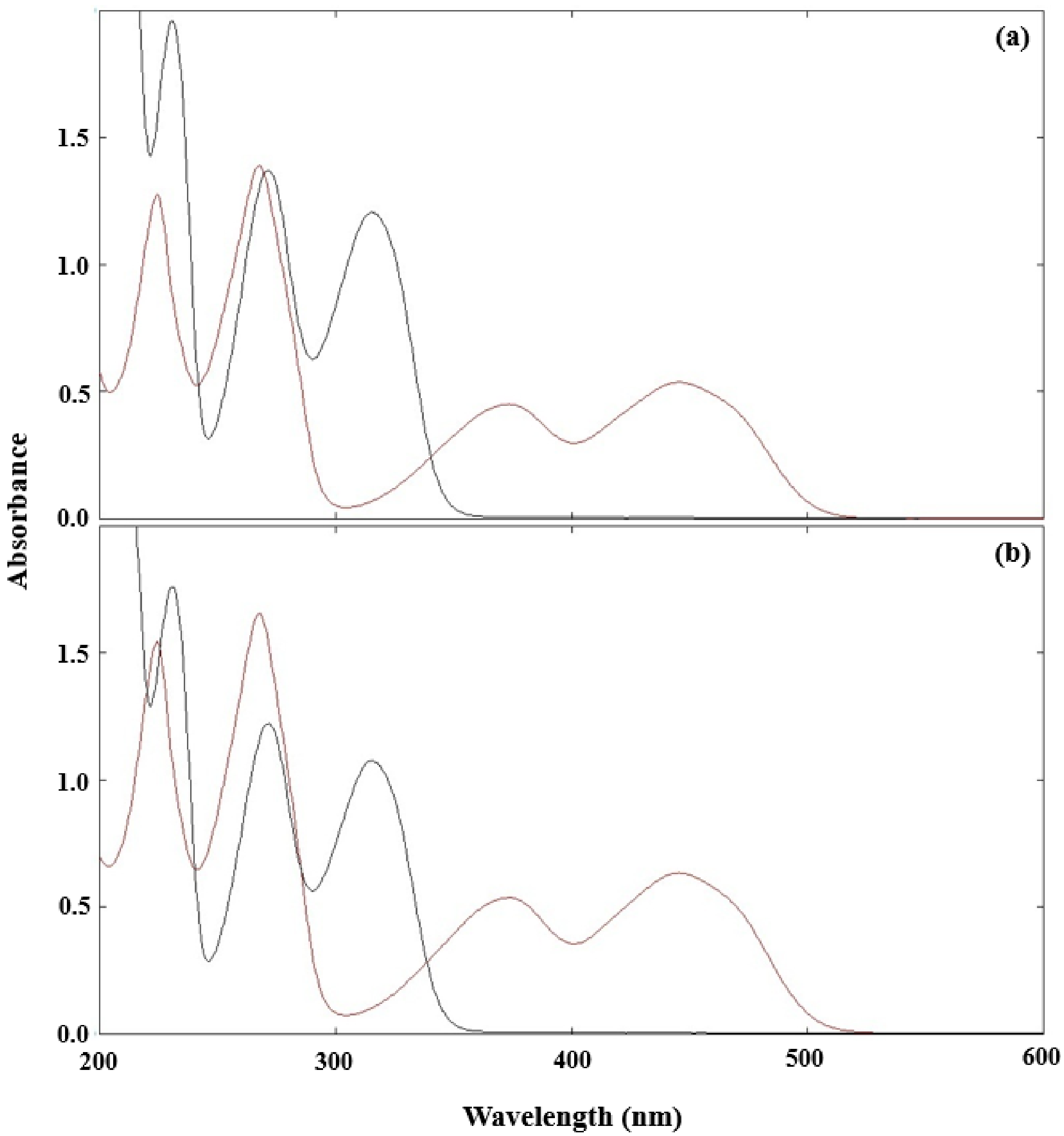

Fig. S5. Absorption spectra of DPZ (1.00×10^{-4} M, black line) and RF (0.50×10^{-4} M, red line) in aqueous solution at pH 2.0 (a) and 7.0 (b).

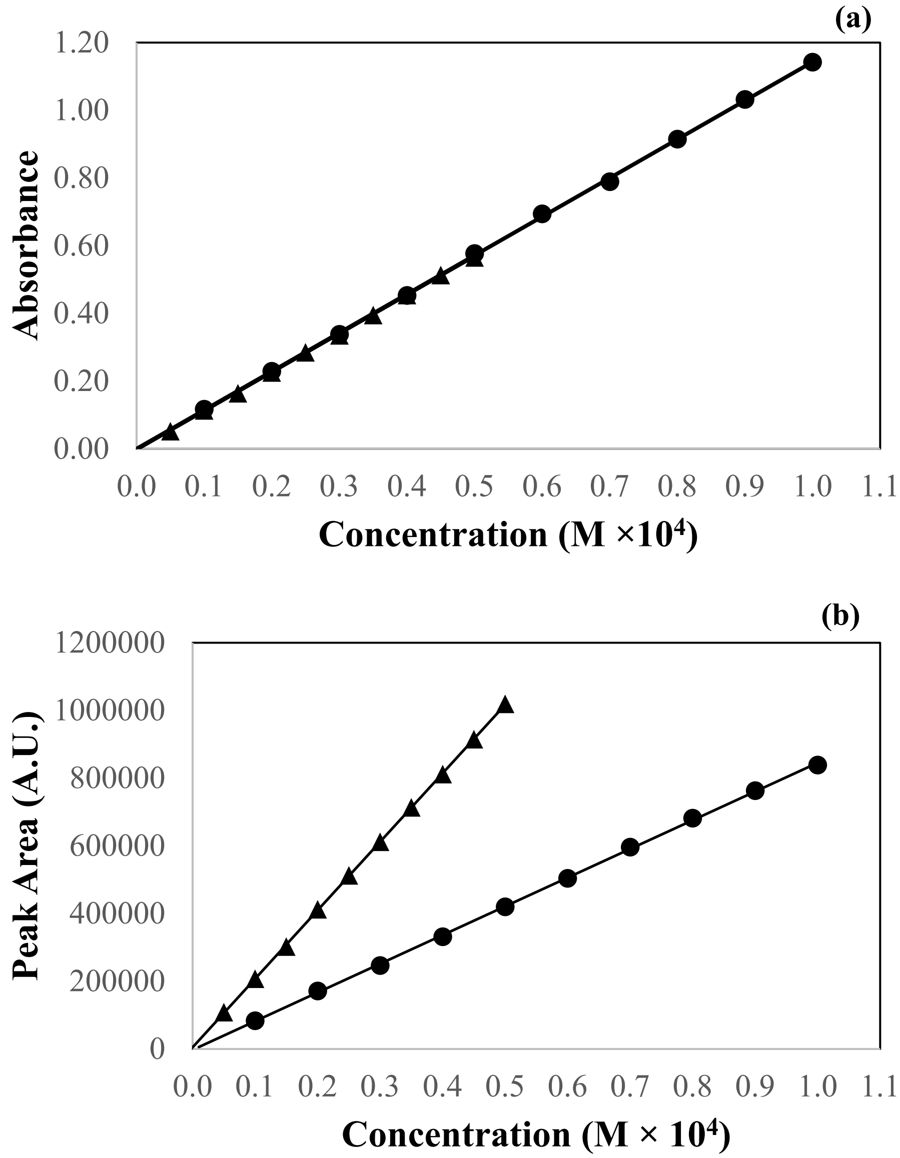

Fig. S6. Calibration curve of DPZ (●) (0.10-1.00 × 10^{-4} M) and RF (▲) (0.05-0.50 × 10^{-4} M) using the proposed two-component spectrometric (a) and HPLC (b) method

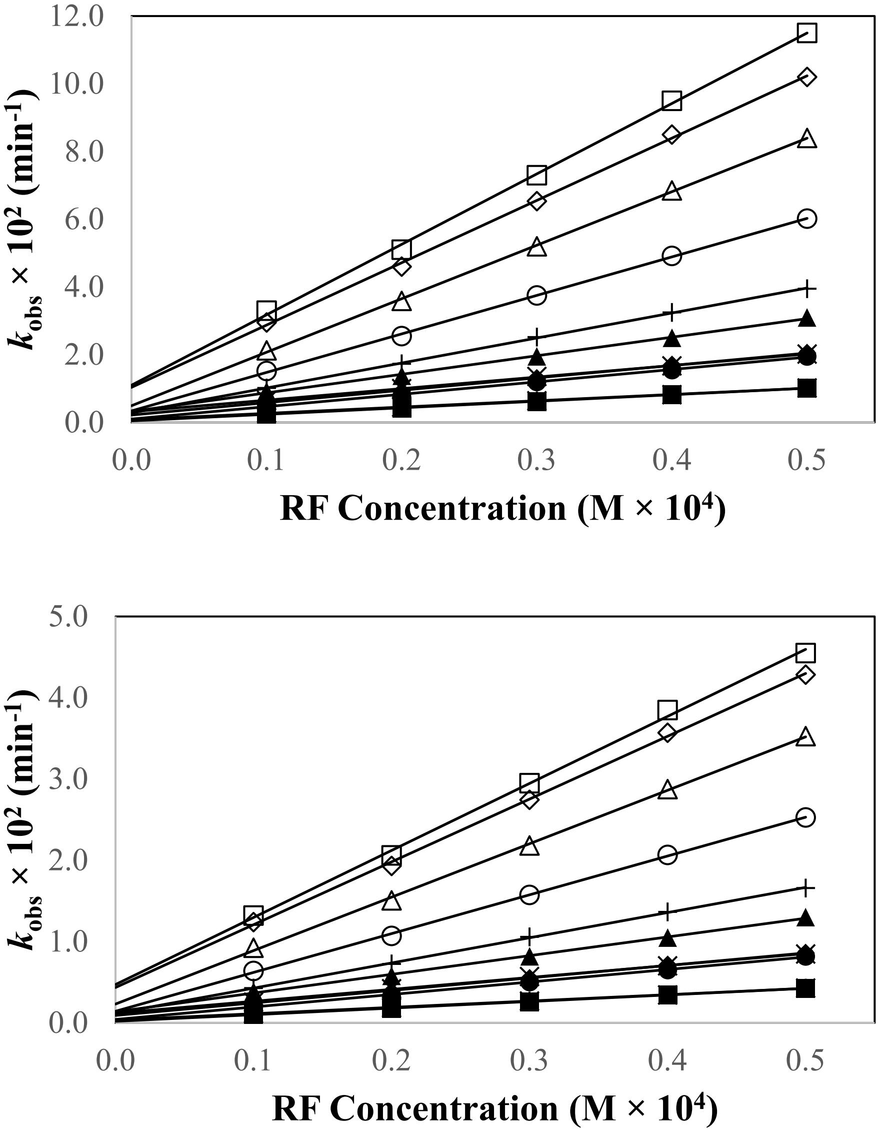

Fig. S7. Second-order plots for the photolysis of DPZ (1.00 × 10^{-4} M) in the presence of RF (0.10-0.50 × 10^{-4} M) in aerobic condition using visible (a) and UV (b) irradiation sources at different pH values: 2.0 (●), 3.0 (▲), 4.0 (♦), 5.0 (■), 6.0 (*), 7.0 (×), 8.0 (+), 9.0 (○), 10.0 (Δ), 11.0 (◊), 12.0 (□).

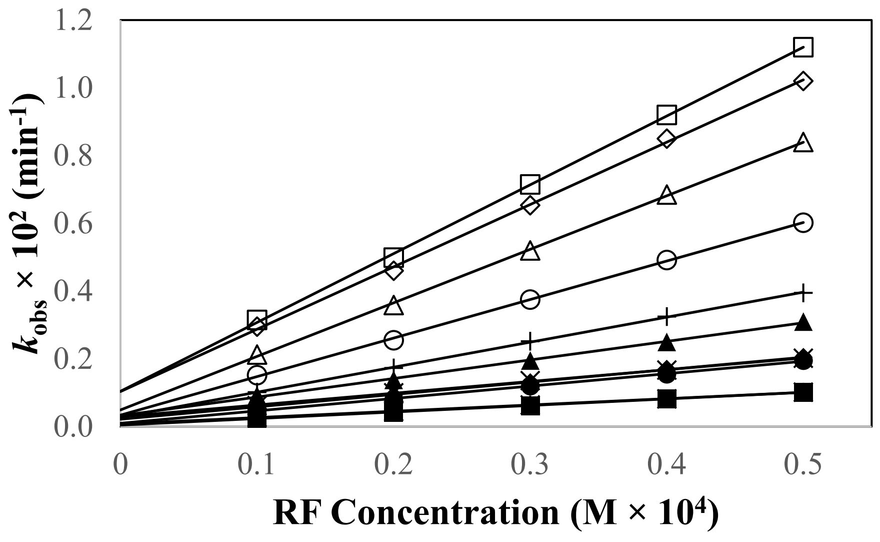

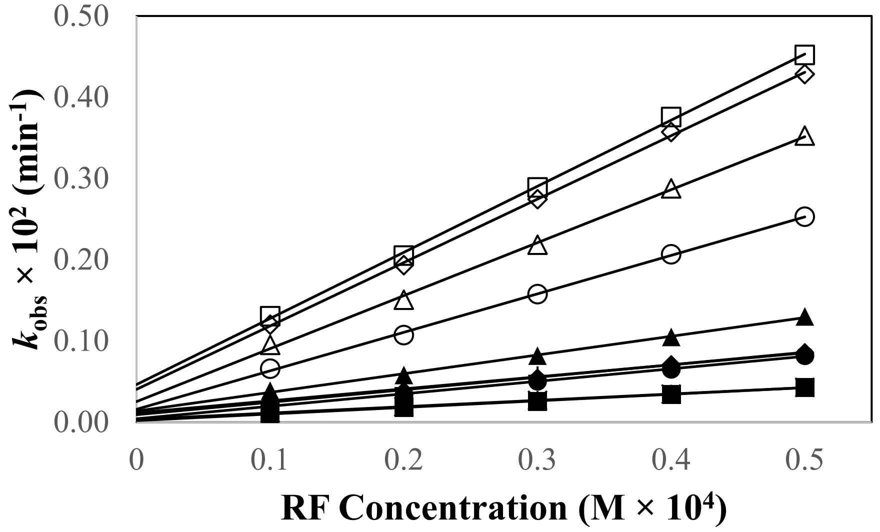

Fig. S8. Second-order plots for the photolysis of DPZ (1.00×10^{-4} M) in the presence of RF ($0.10\text{-}0.50 \times 10^{-4}$ M) in anaerobic condition using visible (a) and UV (b) irradiation sources at different pH values: 2.0 (●), 3.0 (▲), 4.0 (♦), 5.0 (■), 6.0 (*), 7.0 (×), 8.0 (+), 9.0 (○), 10.0 (Δ), 11.0 (◊), 12.0 (□).

Table S1. Accuracy of the proposed two-component spectrometric method for the determination of DPZ and RF

DPZ					RF				
**Added Conc (M×10^4)**	**Found Conc (M×10^4)**	**Recovery (%)**	**Mean Recovery (%)±SD (%RSD)**	**Relative Accuracy Error (%)**	**Added Conc (M×10^4)**	**Found Conc (M×10^4)**	**Recovery (%)**	**Mean Recovery (%)±SD (%RSD)**	**Relative Accuracy Error (%)**
0.100	0.099	99.0		-1.000	0.100	0.100	100.0		-0.332
	0.100	100.0	100.0±1.00 (1.00)	0.000		0.101	101.0	100.3±0.57 (0.57)	0.664
	0.101	101.0		1.000		0.100	100.0		-0.332
0.300	0.301	100.3		0.333	0.300	0.299	99.7		-0.333
	0.300	100.0	100.0±0.30 (0.30)	0.000		0.301	100.3	100.0±0.30 (0.30)	0.333
	0.299	99.7		-0.333		0.300	100.0		0.000
0.500	0.500	100.0		-0.067	0.500	0.500	100.0		0.134
	0.499	99.8	100.1±0.15 (0.15)	-0.266		0.499	99.8	99.9±0.12 (0.11)	-0.067
	0.502	100.1		0.333		0.499	99.8		-0.067

Table S2. Precision of the developed two-component spectrometric method for the determination of DPZ and RF

DPZ					RF				
**Added Conc ($M\times10^4$)**	**Found Conc ($M\times10^4$)**	**Recovery (%)**	**Mean Recovery (%)±SD (%RSD)**	**Relative Accuracy Error (%)**	**Added Conc ($M\times10^4$)**	**Found Conc ($M\times10^4$)**	**Recovery (%)**	**Mean Recovery (%)±SD (%RSD)**	**Relative Accuracy Error (%)**
**Repeatability (Intra-day)**									
0.700	0.699	99.9		-0.114	0.400	0.399	99.8		-0.150
	0.701	100.1		0.171		0.399	99.8		-0.150
	0.700	100.0	100.0±0.08 (0.08)	0.029		0.400	100.0	99.9±0.21 (0.21)	0.100
	0.700	100.0		0.029		0.401	100.3		0.350
	0.699	99.9		-0.114		0.399	99.8		-0.150
**Intermediate (Inter-day)**									
0.700	0.699	99.9		-0.086	0.400	0.400	100.0		0.100
	0.700	100.0		0.057		0.399	99.8		-0.150
	0.700	100.0	99.9±0.05 (0.05)	0.057		0.400	100.0	99.9±0.11 (0.10)	0.100
	0.699	99.9		-0.086		0.400	100.0		0.100
	0.700	100.0		0.057		0.399	99.8		-0.150

Table S3. Accuracy of the proposed HPLC method for the determination of DPZ and RF

DPZ					RF				
**Added Conc ($M\times10^4$)**	**Found Conc ($M\times10^4$)**	**Recovery (%)**	**Mean Recovery (%)±SD (%RSD)**	**Relative Accuracy Error (%)**	**Added Conc ($M\times10^4$)**	**Found Conc ($M\times10^4$)**	**Recovery (%)**	**Mean Recovery (%)±SD (%RSD)**	**Relative Accuracy Error (%)**
0.100	0.099	99.0		-0.669	0.100	0.099	99.0		-0.669
	0.100	100.0	99.7±0.57 (0.57)	0.334		0.100	100.0	99.7±0.57 (0.57)	0.334
	0.100	100.0		0.334		0.100	100.0		0.334
0.300	0.299	99.7		-0.111	0.300	0.300	100.0		0.000
	0.299	99.7	99.8±0.17 (0.17)	-0.111		0.300	100.0	99.9±0.11 (0.11)	0.000
	0.300	100.0		0.223		0.300	100.0		0.000
0.500	0.501	100.2		0.200	0.500	0.499	99.8		-0.067
	0.500	100.0	100.0±0.20 (0.20)	0.000		0.499	99.8	99.9± 0.11 (0.11)	-0.067
	0.499	99.8		-0.200		0.500	100.0		0.134

Table S4. Precision of the developed HPLC method for the determination of DPZ and RF

DPZ					RF				
**Added Conc ($M\times10^4$)**	**Found Conc ($M\times10^4$)**	**Recovery (%)**	**Mean Recovery (%)±SD (%RSD)**	**Relative Accuracy Error (%)**	**Added Conc ($M\times10^4$)**	**Found Conc ($M\times10^4$)**	**Recovery (%)**	**Mean Recovery (%)±SD (%RSD)**	**Relative Accuracy Error (%)**
**Repeatability (Intra-day)**									
0.700	0.699	99.9		-0.114	0.400	0.399	99.8		-0.100
	0.700	100.0		0.029		0.401	100.3		0.401
	0.701	100.1	100.0±0.07 (0.07)	0.171		0.399	99.8	99.9±0.29 (0.29)	-0.100
	0.699	99.9		-0.114		0.398	99.5		-0.351
	0.700	100.0		0.029		0.400	100.0		0.150
**Intermediate (Inter-day)**									
0.700	0.700	100.0		0.000	0.400	0.400	100.0		0.000
	0.700	100.0		0.000		0.401	100.3		0.300
	0.701	100.1	100.0±0.08 (0.08)	0.171		0.399	99.8	100.0±0.20 (0.20)	-0.200
	0.699	99.9		-0.114		0.399	99.8		-0.200
	0.699	99.9		-0.114		0.400	100.0		0.000

Table S5. Robustness of the developed HPLC method for the determination of PD (1.00 ×10^{4} M) and RF (0.50 × 10^{4} M)

Parameters	DPZ			RF		
	Accuracy(%)[a] ±SD	Precision (%RSD)	*t*-test[b]	Accuracy(%)[a] ±SD	Precision (%RSD)	*t*-test[b]
**Wavelength (±2 nm)**						
262	101.4±0.40	0.40	0.75	100.2±0.69	0.69	0.85
266	100.5±0.25	0.25	0.81	101.3±0.45	0.45	0.60
**pH (±0.01 units)**						
2.49	101.4±0.22	0.22	0.99	100.2±0.66	0.66	0.97
2.51	100.3±0.19	0.19	0.84	101.4±0.25	0.25	1.01
**Flow rate (±0.2 ml/min)**						
1.0	100.2±0.30	0.30	1.10	99.85±0.85	0.85	0.82
1.4	100.4±0.36	0.36	0.95	100.3±0.25	0.25	0.94

[a] Accuracy is a mean recovery (%), where n = 5

[b] At a 95% confidence interval, the tabulated value for two degrees of freedom is 2.776, and the values obtained are in the range of 0.60-1.10. Therefore, the $t_{cal} < t_{tab,}$ and there is no difference between the applied changes and the proposed method conditions.

Table S6. Analysis of a synthetic mixture of DPZ and RF using the proposed HPLC method

DPZ				RF			
**Added ($M \times 10^4$)**	**Found ($M \times 10^4$)[a]**	**Recovery (%)**	**RSD (%)**	**Added ($M \times 10^4$)**	**Found ($M \times 10^4$)[a]**	**Recovery (%)**	**RSD (%)**
0.100	0.100	100.0	0.25	0.450	0.450	100.0	0.62
0.200	0.201	100.5	0.19	0.400	0.401	100.3	0.18
0.300	0.299	99.7	0.34	0.350	0.350	100.0	0.88
0.400	0.401	100.3	0.55	0.300	0.300	100.0	0.47
0.500	0.500	100.0	0.17	0.250	0.249	99.6	0.99
0.600	0.602	100.3	0.19	0.200	0.199	99.5	0.84
0.700	0.701	100.1	0.78	0.150	0.149	99.3	0.25
0.800	0.800	100.0	0.49	0.100	0.100	100.0	0.18
0.900	0.899	99.9	0.87	0.050	0.049	98.0	0.33

[a] Values represent the mean of 5 determinations.

Table S7. Mulliken atomic charge analysis of neutral and deprotonated complex

Atom Type	Mulliken charge (Neutral)	Mulliken Charge (Deprotonated)	ΔCharge(Deprot-Neutral)
O	-0.815048	-0.327422	-0.5
O	-0.787568	-0.386455	-0.4
O	-0.793828	-0.425018	-0.4
O	-0.77021	-0.797229	0.0
O	-0.568843	-0.341988	-0.2
O	-0.603616	-0.381297	-0.2
N	-0.856029	0.28143	-1.1
N	-0.486	0.119643	-0.6
N	-0.821298	-0.256243	-0.6
N	-0.891605	0.004089	-0.9
C	0.194895	0.059916	0.1
C	-0.210787	-0.168325	0.0
C	0.120258	0.054119	0.1
C	0.110987	-0.057257	0.2
C	0.412724	-0.062475	0.5
C	0.798308	0.029669	0.8
C	0.177794	-0.152479	0.3
C	-0.310417	-0.137028	-0.2
C	0.042518	-0.209489	0.3
C	-0.010708	0.231687	-0.2
C	0.046568	-0.000766	0.0
C	-0.205102	-0.02292	-0.2
C	-0.0075	-0.106605	0.1
C	0.877816	0.293502	0.6
C	-0.517547	-0.082486	-0.4
C	-0.518995	-0.058004	-0.5
C	0.994932	0.285351	0.7
H	0.163699	0.093635	0.1
H	0.245586	0.102509	0.1
H	0.238945	0.107246	0.1
H	0.186359	0.040726	0.1
H	0.227137	0.05115	0.2
H	0.271617	0.144327	0.1
H	0.150077	-0.080211	0.2
H	0.174468	-0.09064	0.3
H	0.251046	0.11925	0.1
H	0.532836	0.202721	0.3
H	0.468991	0.240031	0.2
H	0.467201	0.263012	0.2
H	0.186351	0.064551	0.1
H	0.196301	0.064935	0.1
H	0.187476	0.050782	0.1
H	0.178564	0.041839	0.1

H	0.169382	0.049537	0.1
H	0.219654	0.051593	0.2
H	0.437515	0.101496	0.3
O	-0.589597	-0.339482	-0.3
O	-0.666044	-0.207298	-0.5
O	-0.658816	-0.193053	-0.5
N	-0.579722	-0.044758	-0.5
C	-0.173001	-0.089278	-0.1
C	-0.308817	-0.075952	-0.2
C	-0.266502	-0.131456	-0.1
C	-0.308702	-0.096754	-0.2
C	-0.32195	-0.096976	-0.2
C	-0.117911	-0.083217	0.0
C	-0.118665	-0.10201	0.0
C	-0.338637	-0.057068	-0.3
C	0.552178	0.335027	0.2
C	0.045781	-0.031865	0.1
C	-0.106395	-0.221316	0.1
C	-0.138747	-0.036539	-0.1
C	-0.28133	-0.171187	-0.1
C	-0.184039	-0.030794	-0.2
C	0.01102	-0.085675	0.1
C	0.371225	0.093569	0.3
C	0.328431	0.002638	0.3
C	-0.206058	-0.087553	-0.1
C	-0.23488	-0.091847	-0.1
C	-0.200434	-0.114858	-0.1
C	-0.20023	-0.103278	-0.1
C	-0.204872	-0.112303	-0.1
C	-0.187402	0.036908	-0.2
C	-0.18401	0.050943	-0.2
H	0.148154	0.082829	0.1
H	0.193022	0.059796	0.1
H	0.164868	0.05329	0.1
H	0.198178	0.08449	0.1
H	0.161304	0.049229	0.1
H	0.174795	0.056365	0.1
H	0.177662	0.056127	0.1
H	0.157937	0.05241	0.1
H	0.12766	0.042477	0.1
H	0.171484	0.047708	0.1
H	0.120867	0.060503	0.1
H	0.190857	0.060032	0.1
H	0.180506	0.086188	0.1
H	0.179666	0.089162	0.1
H	0.14818	0.044967	0.1

H	0.18661	0.053748	0.1
H	0.238089	0.181318	0.1
H	0.267446	0.124001	0.1
H	0.221736	0.10814	0.1
H	0.198842	0.115789	0.1
H	0.207271	0.096243	0.1
H	0.201936	0.11996	0.1
H	0.203727	0.096607	0.1
H	0.175448	0.038005	0.1
H	0.165489	0.06534	0.1
H	0.187557	0.057462	0.1
H	0.174844	0.027192	0.1
H	0.155792	0.035842	0.1
H	0.181701	0.035805	0.1
